# Combined use of conventional and clumped carbonate stable isotopes to identify hydrothermal isotopic alteration in cave walls

**DOI:** 10.1038/s41598-022-12929-4

**Published:** 2022-06-02

**Authors:** Marjan Temovski, László Rinyu, István Futó, Kata Molnár, Marianna Túri, Attila Demény, Bojan Otoničar, Yuri Dublyansky, Philippe Audra, Victor Polyak, Yemane Asmerom, László Palcsu

**Affiliations:** 1 Isotope Climatology and Environmental Research Centre, Institute for Nuclear Research, Eötvös Loránd Research Network, Bem tér 18/c, 4026 Debrecen, Hungary; 2 Institute for Geological and Geochemical Research, Research Centre for Astronomy and Earth Sciences, MTA Centre of Excellence, Eötvös Loránd Research Network, Budaörsi út 45, 1112 Budapest, Hungary; 3 Karst Research Institute, Research Centre of the Slovenian Academy of Sciences and Arts, Titov trg 2, 6230 Postojna, Slovenia; 4 Institute of Geology, University of Innsbruck, Innrain 52, 6020 Innsbruck, Austria; 5 Polytech’Lab, University Nice Côte d’Azur, 930 Route des Colles, Sophia-Antipolis, 06903 Nice, France; 6 Department of Earth and Planetary Sciences, University of New Mexico, Albuquerque, NM 87131 USA

**Keywords:** Geochemistry, Hydrogeology, Petrology

## Abstract

Alteration of conventional carbonate stable isotopes (δ^18^O, δ^13^C) in cave walls has been shown to be a useful tool to identify cave formation driven by deep-seated processes, i.e., hypogene karstification. If combined with a prior information on the paleowater stable isotope composition, further insights can be obtained on the temperature and the source of the paleowater. Clumped isotope composition (Δ_47_) of carbonates is an independent measurement of temperature, and if combined with the conventional stable isotopes, can provide information on the paleowater stable isotope composition. On the example of Provalata Cave (N. Macedonia), we apply for the first time, both conventional and clumped stable isotope analysis, and identify two different isotope alteration trends, reflecting two distinct hydrothermal events: an older, hotter one, where isotope alteration was likely related to isotope diffusion, lowering the δ^18^O values of the carbonate; and a younger one, related to the cave formation by low-temperature CO_2_-rich thermal waters, with dissolution-reprecipitation as the alteration mechanism, causing decrease in δ^18^O values, and unexpected increase in δ^13^C values. The findings are further corroborated by additional insight from optical petrography and cathodoluminescence microscopy, as well as fluid inclusion analysis of secondary calcite crystals related to the cave forming phase.

## Introduction

Hydrothermal caves are one of the main genetic type of caves, a sub-type of hypogene caves, whose origin is related to deep-seated processes (source of acidity and recharge to the soluble rock formation is from depth), contrary to the most commonly found caves whose origin is connected to surface-related processes (i.e., epigene caves; source of acidity and recharge is from the surface)^1–3^. Hydrothermal caves are formed by thermal waters that have generally higher CO_2_ concentrations, and dissolve carbonate bedrock as they cool down along their rising flow path due to inverse relationship of calcite solubility and temperature^4^. At shallower levels of the aquifer, decrease in pressure facilitates CO_2_ degassing, leading to supersaturation and precipitation of calcite^5^. They are generally strongly structure controlled and develop a characteristic suite of cave morphologies and deposits^2,6,7^.

While some active thermal caves are accessible and offer possibility to study some of the related processes occurring in shallower conditions^8,9^, most of the known hydrothermal caves are fossil, and became accessible only after being intercepted by surface erosion^10^. Identification of characteristic morphologies has been the most common approach to identify the hydrothermal origin of caves^11^, however equifinality of some morphological features (e.g., convectional forms such as cupolas, pockets etc.) makes this approach not resolutive. Study of secondary minerals has been proven to be a more reliable approach, with microthermometry and stable isotope composition used to confirm hydrothermal origin and identify the sources of fluids as well as their formation temperature^12–16^.

Although secondary minerals might have been deposited from thermal waters, their hydrothermal origin is not a proof of the primary hydrothermal origin of the cave^14^, unless their relationship to the cave origin can be unambiguously demonstrated^4^. Likewise, the absence of hydrothermal minerals does not exclude a hydrothermal origin of a cave, as they might not precipitate at all, and later modification in shallower settings can modify and overprint the primary hydrothermal features. Isotope alteration of bedrock, an approach developed for and most commonly used in hydrothermal ore-related studies^17–19^, has also been successfully applied to cave wall bedrock. Recent studies, based on alteration of conventional carbonate stable isotope compositions (expressed as δ^18^O and δ^13^C values) in cave walls, have demonstrated a hypogene origin in the presence or absence of related cave minerals^15,20,21^. Furthermore, it has been found that alteration halos do not develop in epigene caves^22^ and this approach can be used to confirm or dismiss hypogene-related cave origin^23^. The most commonly identified alteration in cave walls is lowering of the bedrock δ^18^O values, as a result of the interaction with low-δ^18^O high-temperature meteoric waters, as well as lowering of bedrock δ^13^C values due to interaction with dissolved inorganic carbon (DIC) with lower δ^13^C values^12,14,15,22^.

Recent developments in carbonate clumped isotope thermometry^24^ allow an independent estimate of the mineral formation temperature, that, if combined with the conventional oxygen isotope composition, can be used to reconstruct the paleofluid oxygen composition^25^. Carbonate clumped isotope thermometry has found wide applications, such as in reconstructing metamorphic and exhumation histories of marble formations^26,27^, sediment diagenesis^28,29^, as well as speleothem studies^30–32^. The application of clumped isotope thermometry to subaerially formed speleothems has proven challenging, due to disequilibrium effects related to various controls (e.g., rapid CO_2_ degassing), leading to overestimation of formation temperatures^33,34^. However, subaqueous speleothems, especially slowly precipitating ones, were found most likely to have precipitated in equilibrium, thus providing more reliable results^31,32,35–37^.

Here we expand on the cave wall isotope alteration approach^22^ by applying both clumped and conventional stable isotope analyses on carbonates. On the example of Provalata Cave (N. Macedonia), we show for the first time, that the characteristics of two distinct hydrothermal events causing different cave wall isotope alterations can be identified, the second one responsible for cave formation. We support our findings by additional insight obtained by optical petrography and cathodoluminescence microscopy, as well as fluid inclusion analysis of secondary calcite minerals, and reconstruct the subsequent evolution of the cave.

## Study area

Hypogene karst in Mariovo (N. Macedonia) is found at several localities, at the intersection of low topography and major faults, and is developed mainly in calcite and dolomite marble, that are part of NNW-SSE oriented nappes along the eastern part the Pelagonian massif^38^ (Supplementary Fig. S1). Melnica locality in Mariovo is an output zone of a hypogene karst system, where caves and cave remnants developed in calcite marble, dolomite marble and carbonate breccia, and a small lukewarm Melnica Spring, are found (Supplementary Fig. S2). Provalata Cave is a small fossil cave and the most remarkable example of this system. It was formed in calcite marble in two successive phases, first by CO_2_-rich thermal waters in a phreatic setting, and then by sulfuric acid in a dominantly vadose setting, both leaving characteristic suites of cave morphologies and associated minerals^39^. Sulfuric acid speleogenesis (SAS), that was active at ca. 1.6 Ma, left abundant gypsum deposits formed as a replacement of carbonate bedrock and calcite spar^39,40^. The calcite spar is found as coatings, up to 0.5 m thick, that cap the hydrothermal carbonic phase.

From Provalata Cave we collected two wall-drilled cores^41^ (C2 and C3) that cover cave wall bedrock and calcite coatings, and two additional samples cut from calcite coatings covering the cave wall (PROV03 and PR20). Furthermore, we collected samples along the NNW-SSE nappes to better characterize the primary isotopic composition of the calcite marble. Details of the sampled material are given in the Supplementary Information.

## Results and discussion

### Macroscopic observations

**Figure 1:**
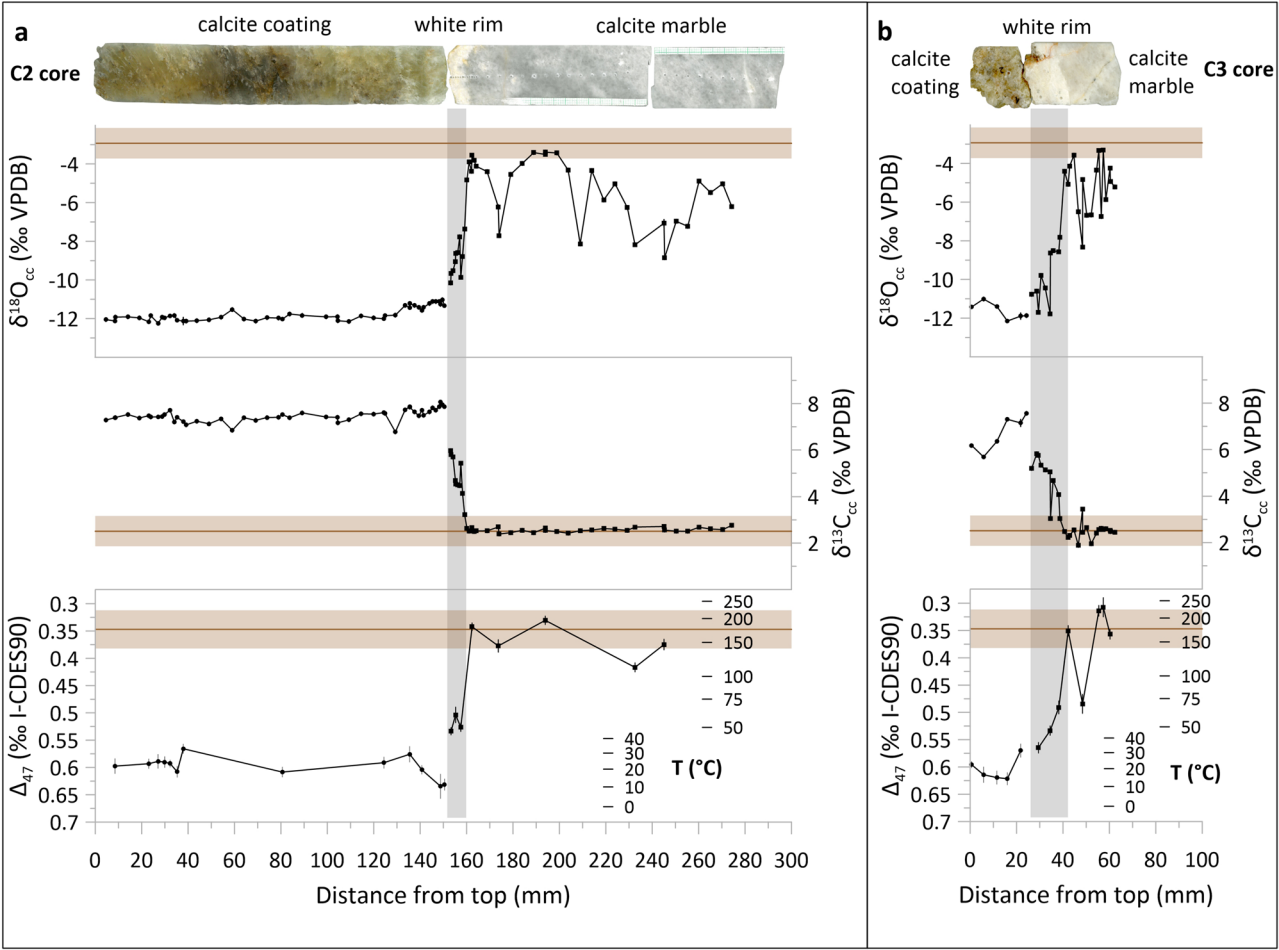
Conventional and clumped stable isotope composition of cores C2 (**a**) and C3 (**b**) from Provalata Cave. Average values and ranges (± 1SD) for calcite marble in the wider area are given by brown lines and brown bands, respectively.

The two cores, C2 and C3, have length of ~ 30 and ~ 6 cm, with calcite marble representing ~ 15 and ~ 4 cm, respectively. In addition to primary grey-colored marble, two distinct types of color alteration are visible: a narrow rim (< 2 cm) with intense white discoloration at the contact of the cave wall and the overlying calcite coating, and a dispersed pale grey discoloration in the inner part (Fig. 1, Supplementary Fig. S5). The thickness of the calcite coatings is significantly different (~ 15 cm and ~ 2 cm), with the one at C3 likely largely dissolved in the subsequent SAS phase.

### Conventional stable isotope composition

**Figure 2:**
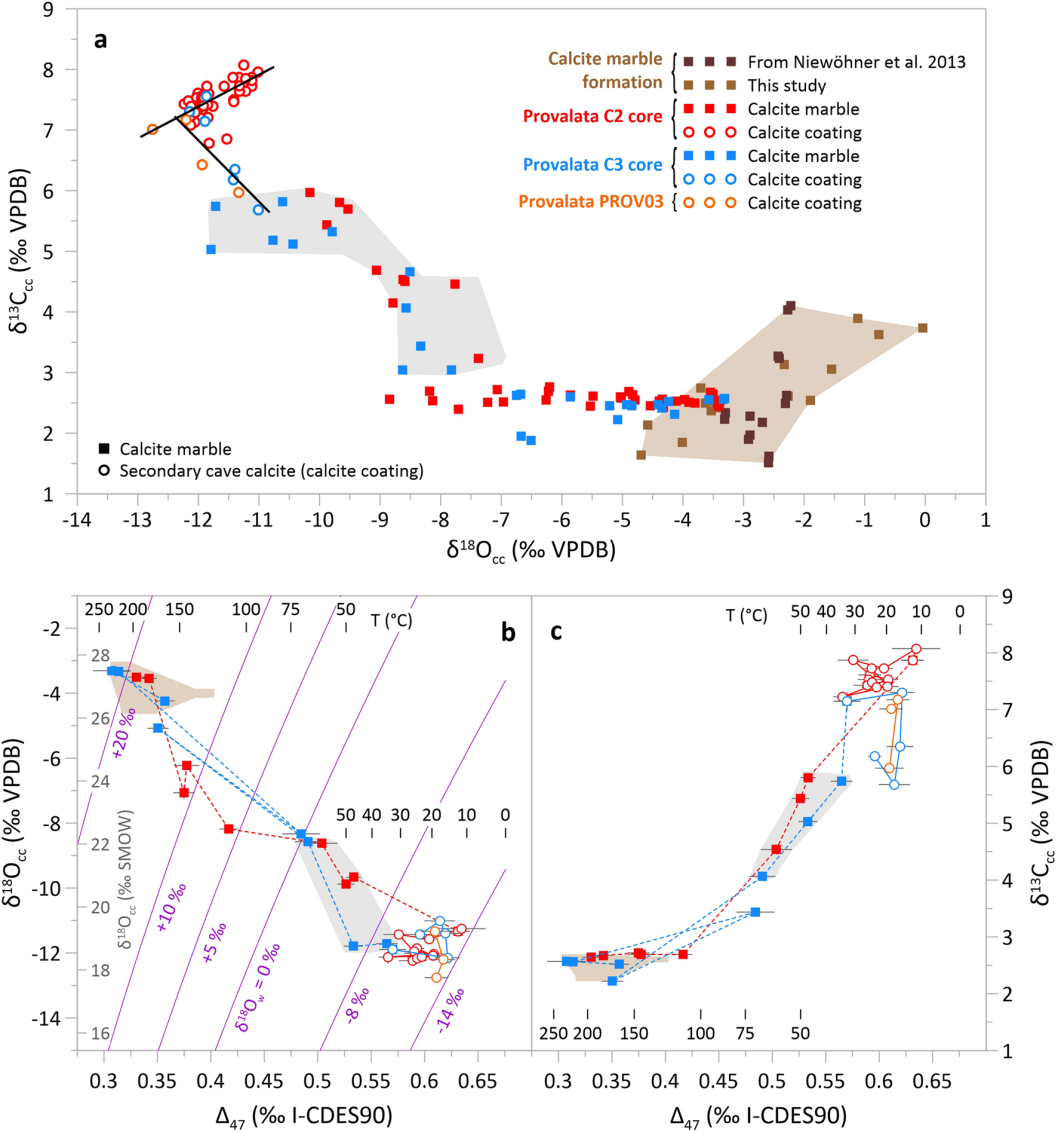
Conventional and clumped stable isotope composition of the studied carbonate samples. (**a**) Conventional stable isotope δ^18^O_cc_ − δ^13^C_cc_ plot. Black lines indicate the two trends of covariation of δ^18^O_cc_ and δ^13^C_cc_ values from Provalata Cave calcite coatings. (**b**) Clumped isotope composition (Δ_47_) vs δ^18^O_cc_. Equilibrium curves based on the equation of Daëron et al.^31^ for a given water stable isotope composition (δ^18^O_w_) are shown in purple. (**c**) Clumped isotope composition vs δ^13^C_cc_. Apparent clumped isotope-based temperatures are also indicated in (**b**) and (**c**). Shaded brown and grey areas show range of values for the unaltered calcite marble and the narrow white rim, respectively.

The grey calcite marble sections in both cores have similar conventional stable isotope composition, with calcite oxygen (δ^18^O_cc_) and carbon (δ^13^C_cc_) isotope values that are within the range of values obtained for samples along the NNW-SSE nappes (Figs. 1, 2). This range of values is essentially the same as what was found from a quarry ~ 30 km to the north^42^, indicating that the grey sections in the cores are isotopically unaltered.

**Figure 3:**
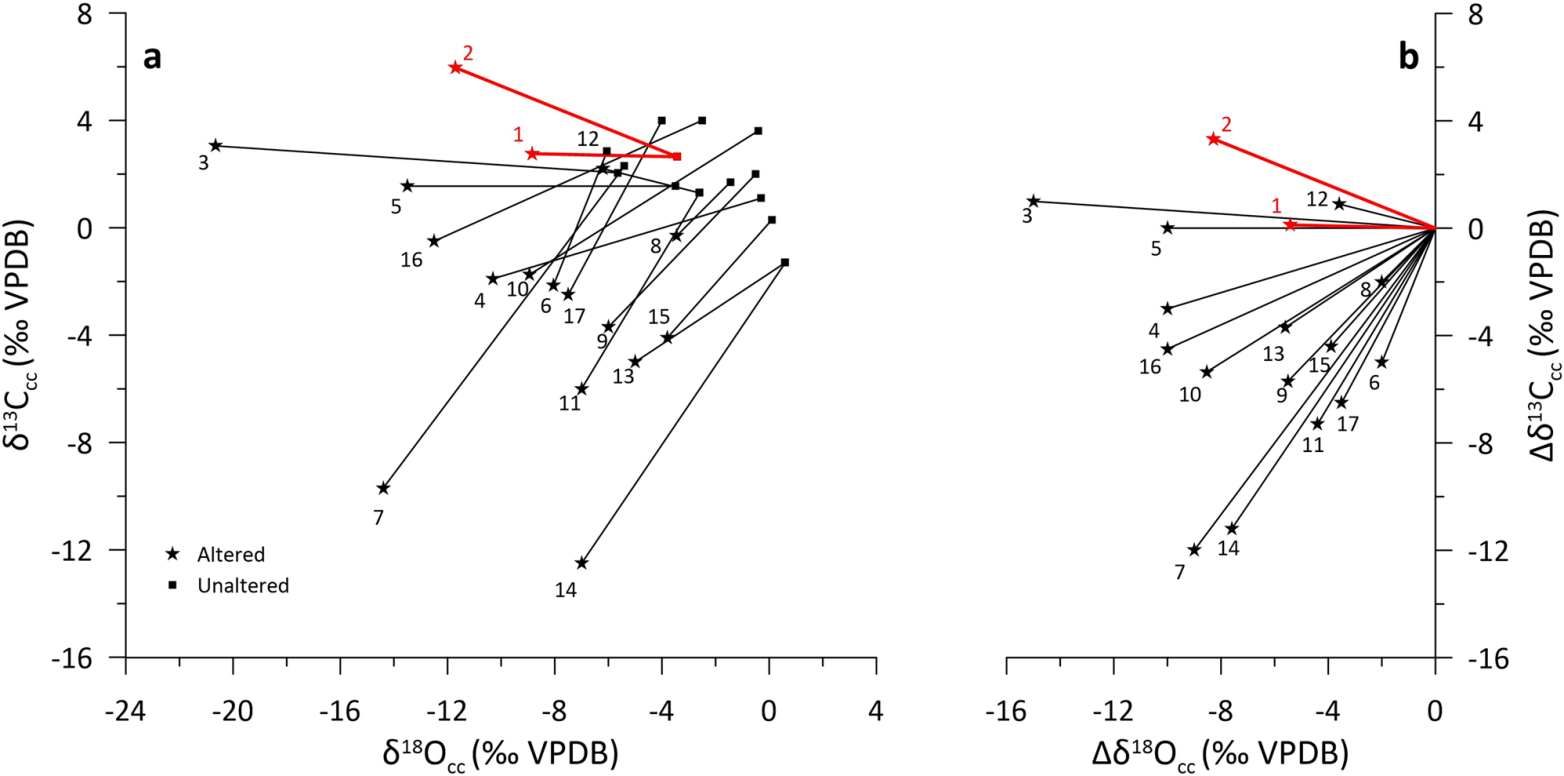
Extent of observed stable isotope alteration in carbonate bedrock walls of hypogene caves. (**a**) Alteration vectors in δ^18^O_cc_ − δ^13^C_cc_ space. (**b**) Magnitude of alteration (Δδ^18^O_cc_ − Δδ^13^C_cc_). 1 and 2—Provalata Cave (this study); 3 to 8—Hypogene caves in Austria^22^ (3—Mitterschneidkar-Durchgangshohle, 4—Entrische Kirche, 5—Stegbachgraben, 6—Obir Caves, 7—Güntherhohle, 8—Marchenhohle); 9—F10 Cave, Dolomites, Italy^23^; 10—Cupp-Coutunn/Promeszutochnaya, Turkmenistan^14^; 11 and 12—Hypogene caves in Crimea, Ukraine^20^; 13 and 14—Movile Cave, Romania^44^, 15—Plavecká jaskyňa, Slovakia^45^; 16 and 17—Carlsbad Cavern, USA^43^.

The two discolored sections of the rock wall have altered δ^18^O_cc_ and δ^13^C_cc_ values that show two distinctive trends. The pale grey parts show no change in δ^13^C_cc_ and decrease in δ^18^O_cc_, with maximum shift of up to − 5.4‰ Δδ^18^O and only + 0.1‰ Δδ^13^C (where ΔX = X_alteredMAX_ − X_unalteredMEAN_, and X is δ^18^O_cc_ or δ^13^C_cc_). The white narrow rim shows change in both δ^13^C_cc_ and δ^18^O_cc_, with Δδ^18^O of − 6.7‰ to − 8.3‰ and Δδ^13^C of + 3.3‰ to + 3.2‰, for C2 and C3, respectively, with values trending towards the isotopic composition of the overlying calcite coatings (Figs. 1, 2). The observed shift to lower δ^18^O_cc_ is commonly found in hypogene caves with reported Δδ^18^O ranging from − 15‰ to − 2‰, reflecting interaction with thermal water of predominantly meteoric origin^14,20,22,23,43–45^ (Fig. 3). However, the δ^13^C_cc_ values are commonly reported to be either unchanged or shifted to lower values, with Δδ^13^C from − 12 to + 1‰, due to interaction with fluid with low δ^13^C DIC values (δ^13^C_DIC_), reflecting predominantly organic-derived carbon^14,20,22,23,43–45^ (Fig. 3). The shift to higher δ^13^C_cc_ values observed in the narrow rims in cores C2 and C3, suggests that the paleowater at Provalata Cave had high δ^13^C_DIC_. This is in agreement with recent geochemical data from nearby lukewarm Melnica Spring, where it was found that up to 54% of the carbon in the DIC is from metamorphic CO_2_ with δ^13^C of + 4.5‰^46^.

The calcite coatings have low δ^18^O_cc_ (mean − 11.8‰) and very high δ^13^C_cc_ (mean + 7.3‰), with a relatively small range of values (Table 1). They show covariation in δ^18^O_cc_–δ^13^C_cc_ space along two distinct trends, one with positive and one with negative slope (Fig. 2).

**Table 1:** Summary statistics of the stable isotope composition of wall bedrock and calcite coatings from Provalata Cave.

Section/sample		δ^18^O_cc_	δ^13^C_cc_	Δ_47_ (‰ I-CDES90)	T (°C)	δ^18^O_w_ (‰ VSMOW)	Δδ^18^O (‰)	Δδ^13^C (‰)
		(‰ VPDB)						
**Calcite marble (C2 + C3 cores)**								
Grey	*min*	− 3.8	2.4	0.307	181	17.7	0	0
	*max*	− 3.3	2.7	0.342	229	21.7		
	***mean***	− **3.5**	**2.6**	**0.323**	**206**	**19.8**		
	*SD*	0.2	0.1	0.016	22	1.8		
	*n*	8		4				
Pale grey	*min*	− 8.8	1.9	0.351	112	5.9	− 5.4	+ 0.1
	*max*	− 3.8	2.8	0.417	171	15.5		
	***mean***	− **5.5**	**2.5**	**0.375**	**147**	**11.8**		
	*SD*	1.3	0.2	0.026	23	3.9		
	*n*	40		5				
White rim	*min*	− 11.8	3.0	0.484	35	− 9.1	− 8.3	+ 3.3
	*max*	− 7.4	6.0	0.565	70	0.2		
	***mean***	− **9.4**	**4.7**	**0.520**	**54**	− **3.9**		
	*SD*	1.2	0.9	0.028	13	3.5		
	*n*	21		7				
**Calcite coatings**								
C2 core	*min*	− 12.2	6.8	0.566	11	− 13.3	/	/
	*max*	− 11.0	8.1	0.635	34	− 9.6		
	***mean***	− **11.8**	**7.5**	**0.599**	**23**	− **11.6**		
	*SD*	0.3	0.3	0.019	6	1.1		
	*n*	49		13				
C3 core*	*min*	− 12.1	5.7	0.570	15	− 13.4	/	/
	*max*	− 11.0	7.6	0.622	33	− 9.7		
	***mean***	− **11.6**	**6.7**	**0.604**	**21**	− **11.6**		
	*SD*	0.4	0.7	0.022	7	1.4		
	*n*	6		5				
PROV03*	*min*	− 12.8	6.0	0.610	17	− 13.3	/	/
	*max*	− 11.3	7.2	0.618	19	− 11.8		
	***mean***	− **12.1**	**6.6**	**0.613**	**18**	− **12.8**		
	*SD*	0.6	0.6	0.004	1	0.9		
	*n*	4		3				
All calcite coatings	*min*	− 12.8	5.7	0.566	11	− 13.4	/	/
	*max*	− 11.0	8.1	0.635	34	− 9.6		
	***mean***	− **11.8**	**7.3**	**0.602**	**22**	− **11.7**		
	*SD*	0.4	0.5	0.019	6	1.2		
	*n*	59		21				

*For samples with available fluid inclusion data, measured δ^18^O_w_ is used. Full dataset is available in the Supplementary Table S5.

### Clumped isotope composition

The clumped isotope compositions follow a similar pattern. The grey calcite marble has the lowest Δ_47_ values (mean 0.323 ± 0.016‰), reflecting highest apparent temperatures (mean 206 ± 22 °C) (Figs. 1, 2, Table 1). Similar values were also found for the calcite marble along the NNW-SSE nappes (Figs. 1, 2). They are in the range of equilibrium blocking temperatures for the carbonate clumped thermometer^24,27,47^, and considering their unaltered δ^18^O_cc_ and δ^13^C_cc_, likely reflect blocking temperatures from the last metamorphic event. Such temperatures are in the range of zircon fission track (FT) closure temperatures, and FT ages from the eastern part of the Pelagonian massif relate them to Late Cretaceous-Paleocene thrusting^48,49^. The altered sections have increased Δ_47_ values showing two distinct clumped isotope compositions, that reflect mean apparent temperatures of 147 ± 23 °C in the pale grey section and 54 ± 13 °C in the white rim (Table 1). The apparent temperatures of the two altered sections, combined with their distinct conventional stable isotope composition (e.g., δ^13^C_cc_), imply that the two alterations were related to two distinct hydrothermal events.

The calcite coatings have the highest Δ_47_ values, reflecting apparent temperatures of 11–34 °C (mean 22 ± 6 °C; Table 1). The onset of calcite deposition in C2 is at 12 °C and in C3 at 33 °C (Table S5), and all calcite coating samples show variation in temperature along their growth (Fig. 1). This variation shows that the evolution of the hydrothermal cave after the onset of calcite deposition was not following a simple cooling and degassing due to shift to shallower conditions, as generally suggested for such systems^4^, but experienced pulsations in temperature, with at least one subsequent heating event of up to ~ 34 °C.

We calculated the water oxygen isotope composition (δ^18^O_w_) of the fluid in equilibrium with calcite for all of the identified sections, using the calcite-water oxygen equilibrium equation^31^, the measured δ^18^O_cc_ and the clumped isotope-based apparent temperature (see also fluid inclusion section). The unaltered calcite marble has δ^18^O_w_ values that are within the range for metamorphic fluids^50^. However, as the apparent temperatures of the unaltered marble represent equilibrium blocking temperatures, their calculated δ^18^O_w_ values are not considered as representative. The pale grey section has lower δ^18^O_w_ values, which are within the range for magmatic fluids^50^. The secondary calcite minerals have the lowest values, reflecting largely meteoric water composition, while the narrow rim has intermediate δ^18^O_w_ values. The apparent temperatures for the pale grey section are within the range of temperatures (100 to 200 °C) of the ore mineralization at the nearby Carlin-type-like Allchar deposit^51^ (Supplementary Fig. S1), that is related to circulation of magmatic fluids with variable contribution of meteoric water, and associated with a ca. 5 Ma volcano-plutonic center of the Kožuf-Voras volcanic system^52–54^.

### Microscopic petrography and alteration mechanisms

**Figure 4:**
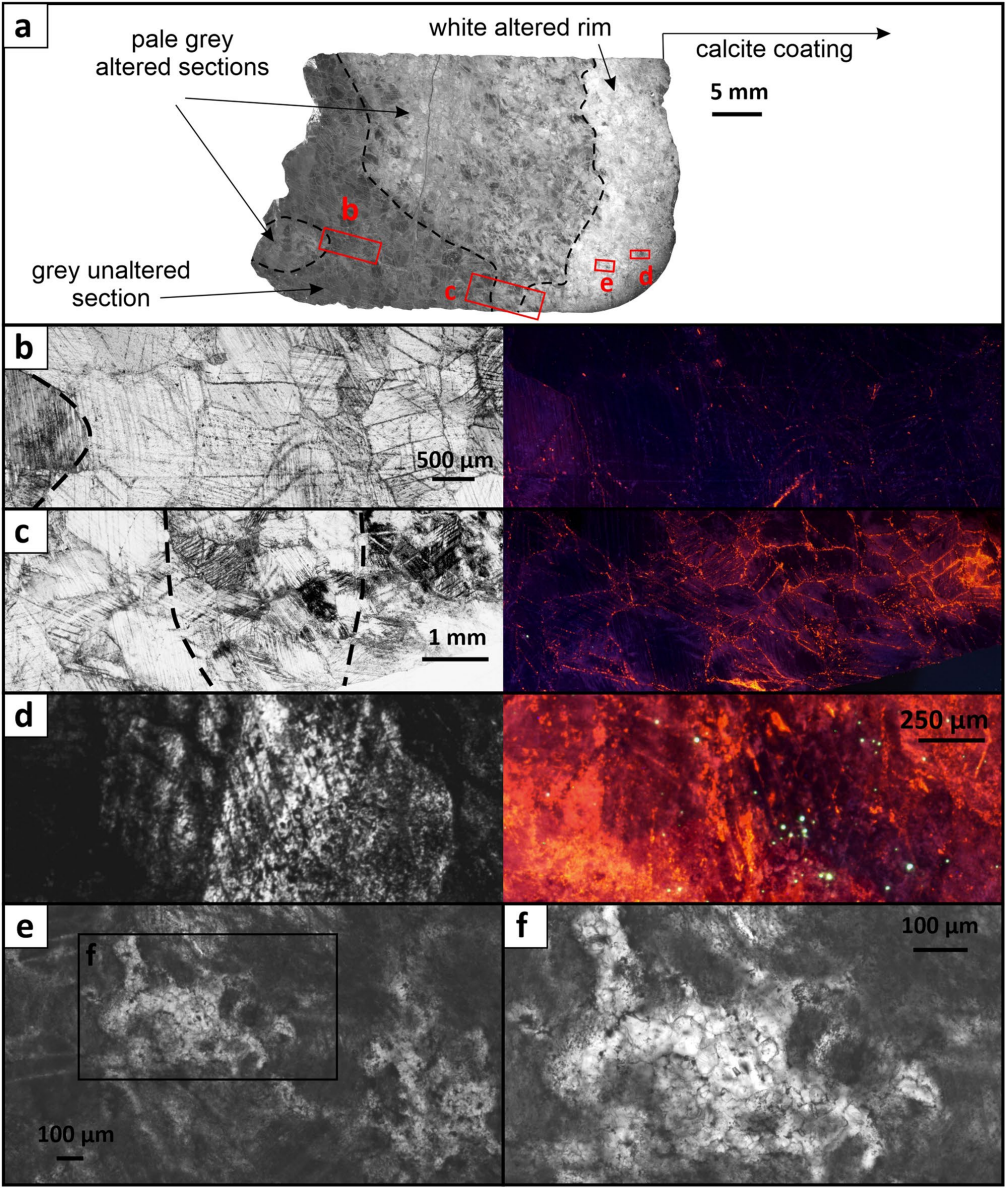
Photomicrographs of C2 core showing different sections of the calcite marble cave wall in plane-polarized transmitted and cathodoluminescent light. (**a**) Overview of the thin section from the outer part of the core with location of the closer examined sections. (**b**) View of the unaltered section of the marble having large (mm size) crystals with dark blue luminescence. (**c**) A cross-section from the unaltered (left) through pale grey (middle) to white rim section, showing dominantly dark blue, violet and orange luminescence, respectively. (**d**) Part of the white rim, with remnants of a large calcite crystal with dark blue luminescence that changes outwards to violet, surrounded by smaller crystals with orange luminescence. (**e**, **f**) Close-up view of the white altered rim section, showing very small calcite crystals filling up pore spaces. An extended version of this figure is found in Supplementary Fig. S6.

The macroscopic and stable isotope observations of the alteration are also supported by the microscopic petrography in both plane-polarized transmitted and cathodoluminescent light (Fig. 4). The grey marble section contains large (up to few mm) calcite crystals, with dark blue luminescence. The pale grey section shows similar size calcite crystals having violet luminescence, found in different positions: throughout the crystal; diminishing inwards from crystal boundaries; or along crystal defects. The narrow white rim section shows remnants of large crystals with dark blue or violet luminescence that are surrounded by a large number of smaller size (up to 50 μm) crystals with orange luminescence, similar to the overlying calcite coating (Fig. 4, Supplementary Fig. S6).

The blue luminescence in the grey marble reflects intrinsic luminescence^55,56^, and supports the primary (unaltered) character of this section as also indicated by the isotopic composition. The similar luminescence of the small calcite crystals in the narrow rim to the one of the overlying calcite coatings suggests deposition from the same (or similar) fluid. This is also supported by the white rim δ^13^C_cc_ and δ^18^O_cc_ values, as they are shifted towards the composition of the overlying calcite coating. A continuous transition between blue, violet and orange cathodoluminescence colors can occur due to the relative and absolute intensity of the intrinsic and orange luminescence^55^. However, in the absence of additional elemental analyses, it is not possible to gain more insight from the cathodoluminescence on the conditions under which calcite precipitation or alteration occurred.

Carbon and oxygen isotopes in calcite can exchange with fluids by either dissolution-reprecipitation, when mineral and fluid are far from chemical equilibrium, or by diffusion, when mineral and fluid are at or close to chemical equilibrium^57^. The exchange might also first proceed by dissolution-reprecipitation until chemical equilibrium is achieved and then by diffusion^58^. Rate of diffusion of carbon and oxygen in minerals is strongly temperature-controlled^57^, but in the presence of water, oxygen diffusion is greatly increased, while carbon diffusion is largely unaffected^59–61^. The alteration of oxygen isotopes with no change in carbon isotopes in the pale grey section, as well as the variation in cathodoluminescence towards the crystal surface, suggest hydrothermal alteration by diffusion. Experimental studies on oxygen isotope diffusion in calcite^61,62^ suggest that the water effect to oxygen diffusion in calcite is primarily a surface process, facilitating oxygen exchange between calcite surface and fluids. However, while water enhances oxygen isotope diffusion at the calcite crystal surface, to alter the clumped isotope composition, reordering of C-O bonds requires bulk mineral increase in oxygen diffusivity^62,63^, which is achieved only at higher temperatures and longer times^25,47^. This implies that dissolution-reprecipitation is the more likely process to explain the alteration of the clumped isotope composition at lower temperatures. Additionally, a combination of increased diffusivity, dissolution-reprecipitation, as well as pressure-induced structural changes can increase the reordering rates^62^. Although our stable isotope data is not detailed enough to investigate variation along a single crystal, the luminescence halo seen along boundaries of some crystal grains, and no apparent sign of dissolution-reprecipitation along the crystals, support the oxygen diffusion interpretation. Thus, the variation of δ^18^O_cc_ and Δ_47_ in the pale grey section most likely reflects the degree of isotopic exchange that occurred between the hydrothermal fluid and the calcite crystals, with alteration progressing further in smaller size crystals and crystals with defects, while creating a halo in larger crystals. The lowest apparent temperature in the pale grey section (~ 110 °C) is found in the sample with the lowest δ^18^O_cc_ values, that likely reflects the temperature of the hydrothermal fluid.

For the narrow rim, the microscopic observations (both under transmitted and cathodoluminescent light) clearly point to dissolution-reprecipitation as the alteration mechanism, where previously formed porosity is filled with secondary calcite that continues further from the cave wall as calcite coating. Thus, the stable isotope composition along the white rim reflects the frequency of secondary calcite minerals, i.e., the linear trends seen in δ^18^O_cc_ − δ^13^C_cc_, Δ_47_ − δ^13^C_cc_, and Δ_47_ − δ^18^O_cc_ space (Fig. 2) reflect the mixing of the isotopic compositions of the secondary calcite and the calcite marble, the latter varying between the compositions of the grey and pale grey sections. Based on the lowest apparent temperature in the white rim, this alteration was caused by a low-temperature (< 35 °C) hydrothermal fluid.

### Fluid inclusions in secondary calcite minerals

**Figure 5:**
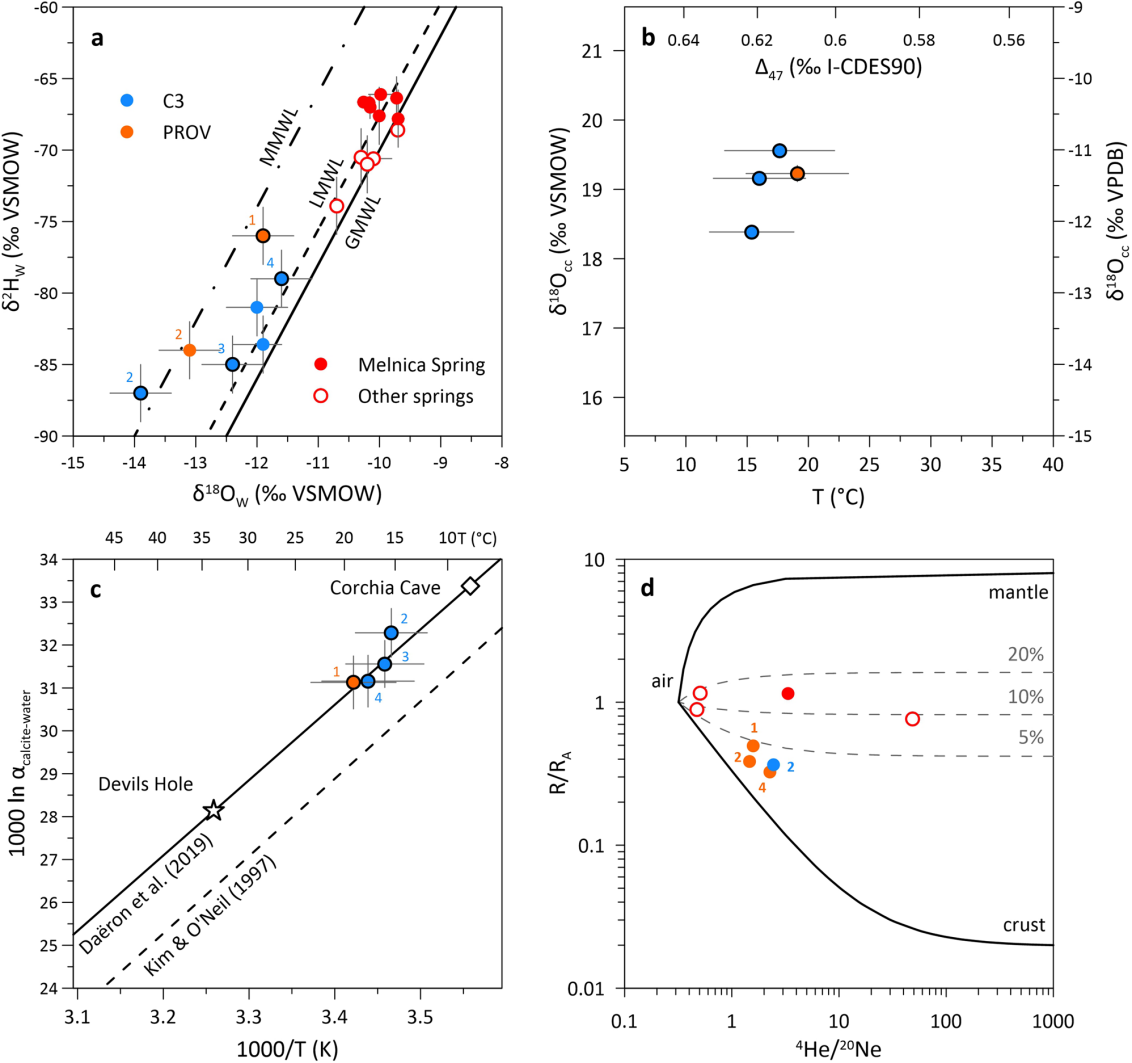
Isotope composition of carbonate and fluid inclusions from Provalata Cave calcite. (**a**) Water stable isotope composition (δ^18^O_w_, δ^2^H_w_). Global (GMWL), Mediterranean (MMWL) and local (LMWL^46^) meteoric water lines are included. Samples for which both carbonate and water stable isotope data are available have black outlines. (**b**) Carbonate stable isotope composition (δ^18^O_cc_, Δ_47_) and clumped based apparent temperatures. (**c**) Calcite-water oxygen fractionation factors for Provalata Cave calcite compared to equilibrium curves of Kim and O’Neil^67^ and Daëron et al.^31^. (**d**) Helium isotope composition (R/R_A_, where R and R_A_ are the ^3^He/^4^He ratio of the sample and air respectively) in a three-component helium mixing plot (atmospheric He: R/R_A_ = 1, ^4^He/^20^Ne = 0.318, crustal He: R/R_A_ = 0.02, ^4^He/^20^Ne = 1000, mantle He: R/R_A_ = 8, ^4^He/^20^Ne = 1000^64–66^). Numbers indicate stratigraphic position of the subsample within the calcite coating sample.

Provalata calcite samples are rich in fluid inclusions that are dominated by primary (intra-crystalline) inclusions, mostly as large size inclusions in palisade crystals, but also as clouds of small ‘thorn’ shaped inclusions (Supplementary Fig. S7). Their water stable isotope composition indicates meteoric origin, with δ^18^O_w_ values ranging between − 13.9‰ and − 11.6‰ and δ^2^H_w_ values between − 87.0‰ and − 76.0‰ (Supplementary Table S1). The data fall between the local and Mediterranean meteoric water lines, with lower values than what was found for the cold and lukewarm springs in the area^46^ (Fig. 5).

Noble gas data show predominance of crustal He in the fluid inclusions, with R/R_A_ values (where R and R_A_ are the ^3^He/^4^He ratio of the sample and air, respectively) of 0.37 to 0.50 and ^4^He/^20^Ne values of 1.5 to 2.4. The three-component (air, crust, mantle^64–66^) helium mixing model (Fig. 5, Supplementary Table S2) shows that the mantle component is low (2–4%) and the atmospheric component ranges from 13 to 22%.

For the samples with combined conventional and clumped carbonate stable isotope data, and fluid inclusion water stable isotope data (i.e., formation T, δ^18^O_w_ and δ^18^O_cc_; n = 4), the calcite-water oxygen isotope fractionation was calculated (Fig. 5). The data fall above the experimental equilibrium line of Kim and O’Neil^67^, and exactly on the empirical equilibrium curve based on slow growing mammillary calcites from Devils Hole and Corchia Cave^31^. The latter is almost identical to the one proposed by Coplen^68^ and to the theoretical prediction by Watkins et al.^69^, indicating that Provalata calcite formed under conditions at or close to thermodynamic equilibrium. Considering this, the paleowater δ^18^O_w_ was calculated for the samples for which no fluid inclusion data were available, using δ^18^O_cc_, Δ_47_-based apparent temperature and the calcite-water equilibrium equation^31^. The calculated δ^18^O_w_ values range from − 13.4‰ to − 9.6‰, with uncertainties of 0.5–1.5‰, due to propagation of larger uncertainties in apparent temperatures.

### Timing of the calcite coating deposition

The calcite coating has been estimated to form during the end of Pliocene or Early Pleistocene^39^. The low U concentrations in C2 samples suggested that the samples are not readily suitable for U–Pb chronology. Three uranium-series analyses were used to suggest that the coating formed during the Early to Middle Pleistocene. If Pliocene in age, both the ^230^Th/^238^U activity and δ^234^U value would measure 1 and 0‰, respectively, which is secular equilibrium in well preserved, unaltered calcite. C2 core exhibits densely crystalline calcite that yielded ^230^Th/^238^U activities of 1.012, 1.028, and 1.027, and δ^234^U values of 10, 22, and 8‰, respectively for samples C2-1, C2-2a, and C2-2b (Supplementary Table S3). The U-series results indicate, given no alteration, that the calcite coating formed after 2.5 Ma. During the subsequent SAS phase, when the cave reached the water table, in addition to gypsum replacing calcite minerals, potassium sulfate minerals (e.g., alunite, jarosite) formed at the contact of sulfuric acid and clay deposits^39^. The 1.6 Ma Ar–Ar age of alunite formed during this phase^39^ thus provides the minimum age limit for the deposition of the calcite coating.

### The evolution of the secondary calcite minerals

**Figure 6:**
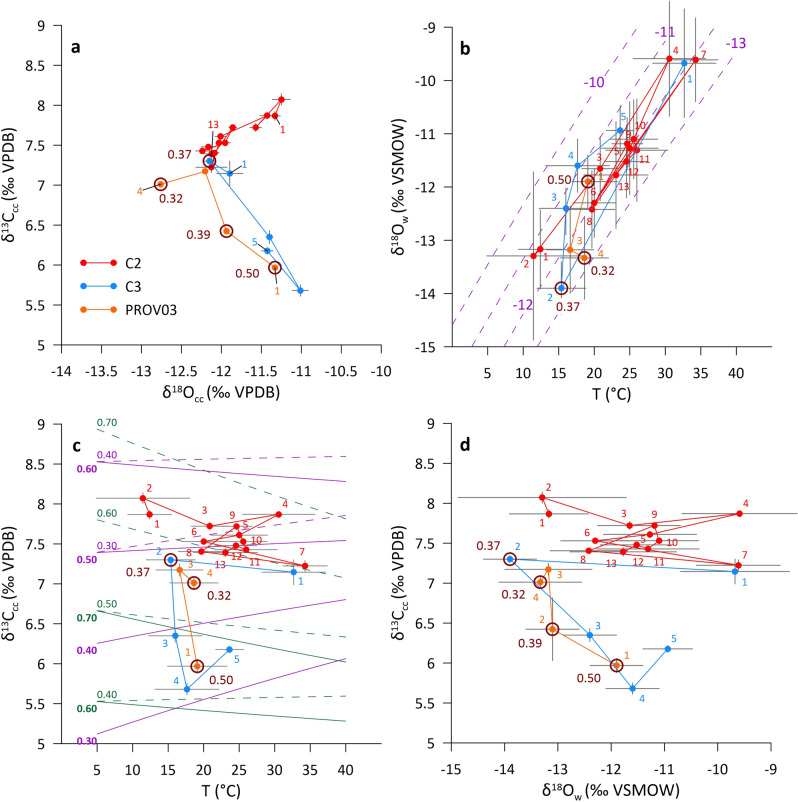
Stable isotope composition of calcite coatings from Provalata Cave. (**a**) Covariation of δ^18^O_cc_ and δ^13^C_cc_ values following generally two lines, one with positive, and one with negative slope. (**b**) Variation of δ^18^O_w_ (from fluid inclusions or calculated) with temperature. Dashed purple lines show equilibrium lines for a given δ^18^O_cc_. (**c**) Variation of δ^13^C_cc_ with temperature. Green and purple full lines show curves of modeled variation of δ^13^C_cc_ with temperature change, with δ^13^C_DIC_ at + 4‰ and + 1‰, respectively, for a selected *f*H_2_CO_3_ (indicated for each curve) and 0.1 mol fraction of carbon in the degassed CO_2_ (X_c_). Closed system calcite precipitation (no degassing) is shown with dashed lines. See Supplementary Information for explanation of calculation and additional modeled curves. (**d**) Variation of δ^18^O_w_ with δ^13^C_cc_. For each sample the stratigraphic sequence of stable isotope data is indicated to observe the trends in stable isotope change. Encircled symbols show noble gas fluid inclusion samples and associated values indicate R/R_A_ values. See text for details.

The calcite coatings in Provalata Cave show small variation in their δ^18^O_cc_ and δ^13^C_cc_ values and a strong covariation that follows two lines: one with positive slope and another with negative slope (Fig. 6). For the samples with positive δ^18^O_cc_ − δ^13^C_cc_ slope, the T-δ^18^O_w_ relationship also follows a positive slope (~ 0.2‰/°C), indicating that the δ^18^O_w_ values are a result of mixing of two fluids: one cold (< 10 °C) with low δ^18^O_w_ (< − 13‰) and another hot (> 35 °C) with higher δ^18^O_w_ (> − 9.5‰). The cold one clearly represents a relatively shallow, meteoric water, while the hot one indicates a deep-seated origin. Magmatic waters have δ^18^O_w_ in the range of + 6‰ to + 10‰ and metamorphic waters in the range of + 5‰ to + 25‰^50^, thus based on the δ^18^O_w_/T slope seen in the samples, and considering magmatic or metamorphic origin, the estimated temperatures of the hot component are between 100 and 200 °C, respectively.

While the water composition is clearly a mixture of two components, the positive correlation in δ^18^O_cc_ − δ^13^C_cc_ space cannot be explained by calcite precipitation due to mixing of two fluids. First, the two fluids will need to be highly saturated in calcite, as mixing of two fluids saturated in calcite at different equilibrium concentrations of Ca^2+^ produces a mixture with lower calcite saturation due to the curvature of the calcite equilibrium line, that will lead to calcite dissolution (i.e., mixing corrosion)^70–72^. Second, the very small change observed in the δ^13^C_cc_ values would require two fluids of such contrasting origin, such as meteoric and deep-seated, to have similar carbon isotopic composition, which is highly unlikely. Furthermore, while mixing of two fluids with similar isotopic composition and contrasting temperatures can produce correlation in δ^18^O_cc_ − δ^13^C_cc_ space, it is unlikely that two fluids will have similar isotopic composition, indicating similar origin, but have contrasting temperature^73^. Thus, it is more reasonable to assume calcite precipitation from one fluid under a temperature effect, then due to mixing of two fluids^73^. This implies that the water mixing occurred prior to calcite precipitation, and likely contributed to the cave passage formation by mixing corrosion.

The calcite that marks the onset of deposition at C2 core has very high δ^13^C_cc_ for a DIC dominated by a meteoric (soil) sourced carbon. The δ^13^C_cc_ depends on the δ^13^C_DIC_, T and pH (distribution of carbon species, mainly H_2_CO_3_ and HCO_3_, having significantly different fractionation with calcite^74^). For such high δ^13^C_cc_ value (+ 7.9‰) at low temperature (12 °C), if the calcite precipitates in isotopic equilibrium from fluid with pH > 6, both δ^13^C_DIC_ and δ^13^C of the CO_2_ (δ^13^C_ext_) should be higher than 0‰, suggesting a deep-seated origin of the CO_2_. At pH above 7 the δ^13^C_DIC_ should be >  + 5.4‰, and δ^13^C_ext_ >  + 7.5‰, with more than 41% of the carbon in DIC coming from carbonate dissolution (see Supplementary Information). However, in such case the alkalinity will be low (Supplementary Fig. S10), and if some soil CO_2_ is present (as expected in shallow meteoric waters), the deep-seated CO_2_ must have even higher δ^13^C, suggesting that the combination of higher pH with such high δ^13^C_DIC_ values is not likely. Thus, it is most likely that the onset of calcite deposition at C2 was from low temperature, slightly acidic fluid, where the water was of meteoric origin, but the CO_2_ was mostly of deep-seated origin. As the subsequent mixing with deep-seated hotter fluid occurred prior to calcite precipitation, this supports the interpretation^46^ that meteoric and deep-seated fluids converged at depth along a fault structure with dominantly metamorphic CO_2_ flux.

Since calcite precipitation is accompanied with temperature change in our samples (Figs. 1, 2, 6), the δ^13^C_cc_ values can be modeled in terms of change in temperature with or without CO_2_ degassing^75^. The modeled curves show characteristic slopes of T-δ^13^C_cc_ for a range of H_2_CO_3_ fractions (*f*H_2_CO_3_) in DIC (Fig. 6; Supplementary Information). At C2, the calcite precipitation follows two lines in T-δ^13^C_cc_ space with similar slightly negative slope, that based on our modeling (Fig. 6), indicates low degassing and lower pH of the fluid. The shift to lower intercept with same slope suggests either lowering of δ^13^C_DIC_ or some variation in the degassing. Similar pattern is also seen in the first part of C3 core. The larger vertical shift in the rest of the samples can be due to δ^13^C_DIC_ change, higher degassing, general shift to higher pH (e.g., lowering of pCO_2_ in the fluid), or a combination. These samples show also larger shift in δ^18^O_w_ (< 2‰). While increase in pH has no direct effect on oxygen isotopes, concurrent increase in pH and δ^18^O_w_ can occur by increased water–rock interaction. However, this is unlikely as the low temperature setting does not favor such large water–rock oxygen isotope exchange.

Another possibility is a change in the primary isotopic composition of both DIC and water. Lowering of δ^13^C_DIC_ of the deep component is a possible explanation (e.g., increase in mantle-derived CO_2_), but increase in mantle-derived fluids cannot explain the observed increase in δ^18^O_w,_ and noble gas fluid inclusion data show minor and relatively constant mantle He component. Variation in the δ^18^O_w_ of the shallow component is very likely, and such increase in values can be due to climate variations (e.g., higher values in warmer and/or drier conditions and vice-versa^76^). Although the effect of δ^18^O_w_ change in the shallow component can be seen in the mixture δ^18^O_w_, the δ^13^C_DIC_ change in the shallow component (carrying a soil δ^13^C signal, e.g., − 25 to − 10‰^76^) will be overprinted by the large fraction of deep-derived CO_2_ (with endogenic δ^13^C signal, e.g., − 7‰ to − 4‰ for mantle, or > 0‰ for metamorphic CO_2_^50,77^. If the deep-sourced CO_2_ flux decreases, pCO_2_ will decrease, leading to an increase in pH and lowering of the δ^13^C_cc_, but also relative increase in the shallow component carbon fraction, and further lowering of δ^13^C_DIC_ and δ^13^C_cc_. This is supported by the noble gas fluid inclusion data, where δ^13^C_cc_ decrease is accompanied with R/R_a_ increase and ^4^He/^20^Ne decrease (Fig. 6), that reflects concurrent increase of the atmospheric He component (from 13 to 22%) and decrease of the crustal He component (from 84 to 76%), with constant minor mantle He component (2–4%).

In other words, climatically controlled variation in the isotopic composition of the cold end-member (δ^18^O_w_, δ^13^C_DIC_) is seen in the mixture δ^18^O_w_ but generally masked in the mixture δ^13^C_DIC_ by the high amount of deep-sourced CO_2_. Thus, the offset from the positive δ^18^O_cc_ - δ^13^C_cc_ covariation in Provalata calcite reflects a phase of lowering of deep CO_2_ contribution.

### The evolution of the hydrothermal cave system: a brief summary

**Figure 7:**
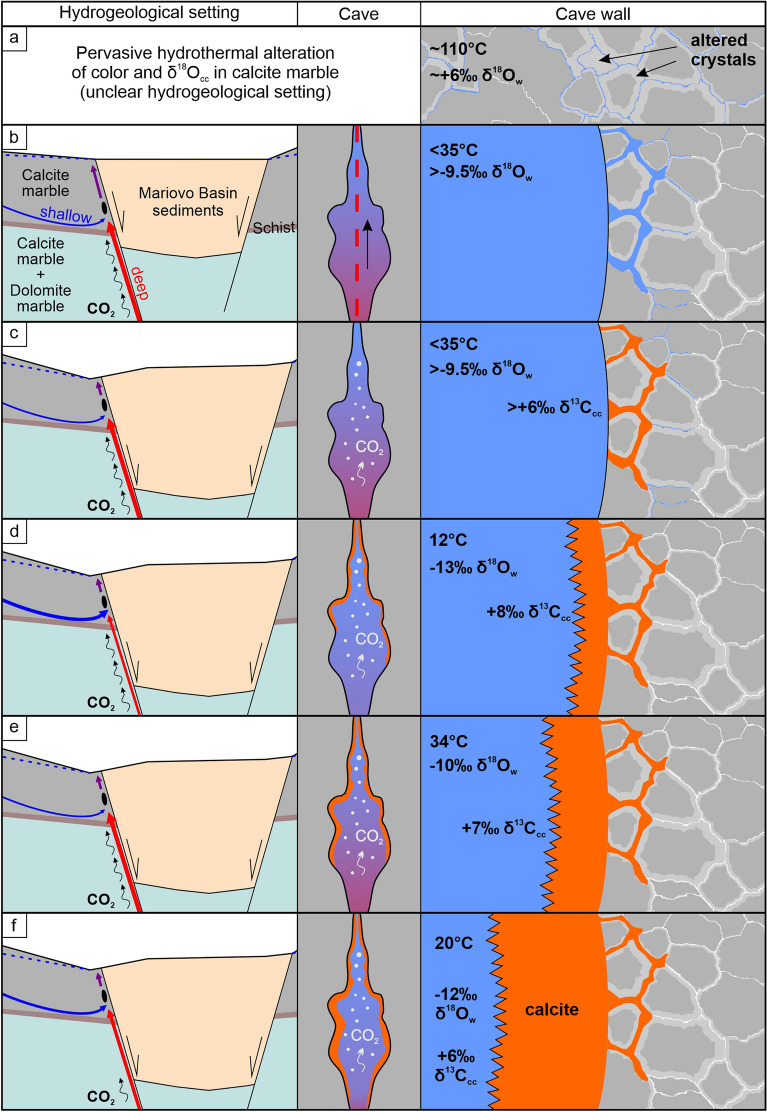
Conceptual model of the evolution of the studied hydrothermal phases in Provalata Cave with associated alteration mechanisms and calcite deposits presented schematically at three scales: hydrogeological setting, cave and cave wall. The thickness of the blue and red groundwater flowlines in each phase indicates the relative influence of the shallow epigene and deep hypogene component, respectively. (**a**) Pervasive alteration of color and δ^18^O_cc_ in calcite marble by hydrothermal water (~ 110 °C) with oxygen diffusion along crystal boundaries as the most likely alteration mechanism. The hydrogeological setting is unclear. (**b**) Development of cave passages along high-angle fractures due to cooling of low-temperature (< 35 °C) thermal waters rich in metamorphic CO_2_, likely enhanced by mixing corrosion. Porosity increases perpendicularly to the cave wall. (**c**) Shift to shallower depth leads to CO_2_ degassing and calcite precipitation, filling up pore spaces. (**d**) Large decrease in temperature and δ^18^O_w_, indicating increased influence of the shallow groundwater component. Calcite with high δ^13^C_cc_ values precipitates outward from the cave wall. (**e**) Increase in temperature and δ^18^O_w_, likely related to nearby volcanic activity, leads to precipitation of calcite with lower δ^13^C_cc_. (**f**) Temperature and δ^18^O_w_ decrease. Lowering of pCO_2_ leads to decrease in calcite δ^13^C_cc_.

The grey calcite marble of the cave walls reflects primary isotopic composition, that was first altered in color and isotopic composition by hydrothermal water (~ 110 °C) of magmatic or mixed magmatic-meteoric origin (Fig. 7). It was likely close to chemical equilibrium with the rock, and caused only lowering of δ^18^O_cc_, probably by oxygen diffusion between water and rock. The alteration was pervasive, and appearing not bound to a major fissure, although the hydrogeological setting under which it operated is unclear. This event probably corresponds to an earlier volcanic activity of the Kožuf-Voras system (6.5–1.8 Ma^52–54^). The second alteration (narrow rim along cave walls), is related to the cave-forming phase, when convectional features developed along high-angle fractures of a NW–SE oriented fault zone^39^, related to Late Miocene-Quaternary extension^48,49^. Cave passages formed by dissolution and enlargement of fractures during cooling of rising low-temperature (< 35 °C) thermal waters (of mixed meteoric and deep-seated origin), rich in metamorphic CO_2_, likely enhanced by mixing corrosion. As dissolution progressed perpendicular to cave walls, porosity was increased in the first few centimeters. Cooling continued, but the system shifted to shallower settings, forcing CO_2_ degassing and calcite precipitation, first filling up pore spaces, and progressing into thick calcite coatings. The thermal evolution and δ^13^C_cc_ values of the coatings suggest that after the initial cooling, another heat pulse followed, and variation in pCO_2_ controlled the variation in δ^13^C_cc_. The calcite deposition took place between 2.5 Ma and 1.6 Ma and is likely connected to the youngest volcanic activity of the Kožuf-Voras system (3.0–1.8 Ma), focused in its southwestern part^52–54^, close to the study site (Supplementary Fig. S1). It is possible that both alteration phases are part of the long-term evolution of the same hydrothermal system, that first commenced at deeper settings with pervasive alteration, and then evolved along newly formed extension-related high-angle faults with high CO_2_ flux, creating cave passages.

## Conclusions

We show that by combined use of conventional and clumped stable isotope analysis of carbonates in the study of cave wall alteration by thermal waters we can better constrain the character of the isotopic alteration, as well as unambiguously identify different alteration events.

On the example of Provalata Cave we demonstrate the primary isotopic character of the unaltered calcite marble, and identify two distinct hydrothermal phases causing different alterations. The older one was pervasive with flow along smaller structures and crystal boundaries, causing lowering of δ^18^O_cc_ values likely due to diffusion-controlled isotope exchange with fluid at or close to chemical equilibrium with the rock, at higher temperature (~ 110 °C). The second one was related to cave formation by fluid flow along a fracture, i.e., progressed perpendicularly inward from the fracture (cave wall), and proceeded with dissolution-reprecipitation-controlled alteration by lower-temperature fluids (< 35 °C) that transitioned from being calcite-undersaturated (dissolution) to calcite-saturated (precipitation).

While most of the examples of cave wall isotope alteration reported in the literature show lowering of δ^18^O_cc_ and either unchanged or lowered δ^13^C_cc_, our findings show a rare example of isotope alteration with lowered δ^18^O_cc_ and significantly increased δ^13^C_cc_. We expect that such case is not a unique one and can be expected at sites with high metamorphic CO_2_ flux. We also show a non-linear thermal evolution of the hydrothermal cave system, suggesting that simple cooling, as usually assumed for such systems, is likely simplifying the evolution, especially for karst systems related to volcanic areas.

## Methods

### Stable isotope analysis of carbonates

Conventional and clumped stable isotope analyses of carbonates were carried out at the Isotope Climatology and Environmental Research Centre (ICER), Institute for Nuclear Research (ATOMKI), Debrecen.

Conventional carbon and oxygen stable isotope analyses were carried out on hand-drilled carbonate powders with an automated GASBENCH II sample preparation device (phosphoric acid digestion at 72 °C) attached to a Thermo Finnigan Delta^PLUS^ XP isotope ratio mass spectrometer (IRMS)^78^. Conventional stable isotope results are expressed as δ^18^O and δ^13^C values relative to Vienna Pee-Dee Belemnite (VPDB). The precision of the measurements is ≤ 0.08‰ for δ^13^C and ≤ 0.1‰ for δ^18^O.

Clumped isotope analysis was done on a Thermo Scientific 253 Plus IRMS, after phosphoric acid digestion at 70 °C using a Thermo Scientific Kiel IV automatic carbonate device. Each carbonate sample measurement consisted of at least 10 replicate analyses of 100–120 μg aliquots that were measured alongside carbonate standard samples (ETH1, ETH2, ETH3, ETH4, and IAEA-C2) with assigned values^79,80^. Data evaluation was done on the Easotope application^81^, with the CO_2_ clumped ETH PBL replicate analyses method and IUPAC parameters^82–85^. Δ_47_ results are given in the I-CDES90 scale^80^, and apparent temperatures in °C were calculated based on the Δ_47_-temperature calibration from Anderson et al.^86^, with temperature uncertainties propagated from the 1σ standard error (SE) of the Δ_47_ value. Simultaneously during clumped isotope analysis, on the same samples, conventional carbonate stable isotope composition was also determined.

### Petrography

Double-polished sections for petrographic observations were prepared employing low-speed precision sawing and polishing, which minimize thermal and mechanical stresses on the samples. Fluid inclusion observations of secondary calcite were carried out on a transmitted-light microscope (Nikon Eclipse E 400 POL) at the Institute of Geology, University of Innsbruck and petrographic observations of calcite marble from cave walls were carried out on a transmitted-light microscope (Olympus BX53 equipped with Olympus DP23 camera) at ICER, ATOMKI, Debrecen. Additionally, cathodoluminescence studies were carried out at the Karst Research Institute ZRC SAZU, Postojna, on a Technosyn® cold cathodoluminescence luminoscope (CITL CL8200 MK4) with a 14 kV electron beam and an electron current of 350–400 μA, mounted on a Nikon Eclipse E600 biological petrographic microscope. The thin sections, examined in transmitted light and cathodoluminescent light, were photographed using a Nikon Coolpix 990 digital camera.

### Fluid inclusion

Fluid inclusion analyses were carried out at three laboratories. Water stable isotope composition was determined at the Institute of Geology, University of Innsbruck, and at the Institute for Geological and Geochemical Research (IGGR), Budapest. Noble gas isotope composition (^3^He, ^4^He, ^20^Ne, ^22^Ne) was determined at ICER, ATOMKI.

At Innsbruck sample PR20 was crushed in a heated crushing cell^87^, and after cryogenic focusing, the water was transported by He flow into the high-temperature reactor of the TC/EA unit (ThermoFisher) and pyrolyzed into H_2_ and CO at 1400 °C. The evolved gases were separated in a chromatographic column and analyzed using a Thermo Fisher Delta V Advantage IRMS. Detailed description of the method can be found in Dublyansky and Spötl^88^. The remaining samples were analyzed at the IGGR in Budapest. Sample chips of about 0.5–1 g were crushed under vacuum in stainless steel tubes, after which the released water was purified by vacuum distillation and introduced into a Los Gatos liquid water isotope analyzer (LWIA-24d). Details of the method are described in Demény et al.^89^. Results are expressed as δ^2^H and δ^18^O relative to Vienna Standard Mean Ocean Water (VSMOW). Analytical precision is 0.5‰ for δ^18^O and better than 1.5‰ (Innsbruck) or 2‰ (IGGR) for δ^2^H.

For noble gas isotope analyses, 1.3 to 2.3 g of calcite samples were loaded into stainless-steel holders with a magnetic ball and baked at ~ 60 °C for 10–12 h in vacuum before the measurements. Gas was extracted by single-step crushing (~ 100 strokes) at room temperature (22 °C). The relatively low number of strokes was applied for each measurement in order to avoid significant contribution of an in-situ component. Helium isotope abundances and ratios were determined by a HELIX- SFT mass spectrometer, whereas a VG-5400 mass spectrometer was used for neon. The analytical procedures are described in more detail in Papp et al.^90^ and Molnár et al.^91^.

### Uranium-series

Three small pieces of densely crystalline calcite coating (core C2) were dissolved in 15 N HNO_3_, doped with a ^229^Th, ^233^U, and ^236^U mixed spike, and cleaned and separated U and Th in anion exchange resin column chemistry following the methods of Asmerom et al.^92^. Measurements were made on a Thermo-Neptune multicollector inductively coupled plasma mass spectrometer at the Department of Earth and Planetary Sciences, University of New Mexico. U and Th were measured separately as static runs where all isotopes are measured in faraday cups except for ^230^Th and ^234^U, which were measured in a secondary electron multiplier. Efficiency between the cups and multiplier were determined using an in-house Th standard and the U standard NBL-112. Decay constants and a detailed description of the uranium-series methods are in Cheng et al.^93^. The measured δ^234^U value for standard NBL-112 (CRM-112a) is -38.5‰, where δ^234^U = [(^234^U_A_/^238^U_A_) – 1] × 1000, and A refers to activity.

### Modeling of carbon isotope composition

To model the δ^13^C_cc_ values in terms of calcite precipitation due to change in temperature with or without CO_2_ degassing, we used the equation of Zheng^75^ with a modification, that instead of considering two extreme cases of HCO_3_-dominant or H_2_CO_3_-dominant fluid, for which only fractionation of calcite-HCO_3_ or calcite-H_2_CO_3_ is used, respectively, we select a set of fractions of H_2_CO_3_ in the DIC of the fluid (where DIC = *f*H_2_CO_3_ + (1-*f*) × HCO_3_), that itself reflects fluid pH at a given T. For a selected δ^13^C_DIC_, range of temperatures and *f*H_2_CO_3_, first we calculate the δ^13^C of the CO_2_ in equilibrium with the fluid (δ^13^C_CO2_) and then we calculate δ^13^C_cc_ values using the Rayleigh model equation of Zheng^75^.

To model the fluid composition from which calcite can precipitate in isotopic equilibrium recording temperature and δ^13^C_cc_ as found for the onset of calcite deposition in C2 core, first we calculate the fractions of H_2_CO_3_ and HCO_3_ in PHREEQC Version 3 software^94^ for a range of pH values, with a fixed temperature and calcium and DIC concentrations determined by forcing charge balance to the solution at fixed calcite saturation index. Then we calculate the δ^13^C_CO2_ and δ^13^C_DIC_, and using the modeled chemical composition for dissolution of only calcite or calcite and dolomite, we determine the isotopic composition of the carbon from CO_2_ not accounted for by dissolution of carbonate rocks (external CO_2_; δ^13^C_ext_).

Detailed explanation of the calculations is given in the Supplementary Information.

### Consent to publish

Consent was obtained from the identifiable person in Fig. S4b (Marjan Temovski) to publish image in an online open access publication.

## Supplementary Information

Supplementary Information.

## Data Availability

All data generated or analysed during this study are included in this published article and its Supplementary Information file.
