# Supplementary material for: Combined use of conventional and clumped carbonate stable isotopes to identify hydrothermal isotopic alteration in cave walls
Source: Sci Rep. 2022 Jun 2;12:9202. doi: 10.1038/s41598-022-12929-4 (PMC9163031; doi:10.1038/s41598-022-12929-4)
Supplement: Supplementary file 1 — Supplementary Information. [file 41598_2022_12929_MOESM1_ESM.pdf]

Supplementary Information to:

Combined use of conventional and clumped carbonate stable isotopes to identify hydrothermal isotopic alteration in cave walls

Marjan Temovski[1*], László Rinyu[1], István Futó[1], Kata Molnár[1], Marianna Túri[1], Attila Demény[2], Bojan Otoničar[3], Yuri Dublyansky[4], Philippe Audra[5], Victor Polyak[6], Yemane Asmerom[6], László Palcsu[1]

[1]Isotope Climatology and Environmental Research Centre, Institute for Nuclear Research, Eötvös Loránd Research Network, Bem tér 18/c, 4026 Debrecen, Hungary

[2]Institute for Geological and Geochemical Research, Research Centre for Astronomy and Earth Sciences, MTA Centre of Excellence, Eötvös Loránd Research Network, Budaörsi út 45, 1112 Budapest, Hungary

[3]Karst Research Institute, Research Centre of the Slovenian Academy of Sciences and Arts, Titov trg 2, SI-6230 Postojna, Slovenia

[4]Institute of Geology, University of Innsbruck, Innrain 52, 6020 Innsbruck, Austria

[5]University Nice Côte d’Azur, Polytech’Lab, 930 route des Colles, 06903 Sophia-Antipolis, Nice, France

[6]Department of Earth and Planetary Sciences, University of New Mexico, Albuquerque, NM, 87131, USA

*corresponding author: temovski.marjan@atomki.hu

Location of the study area

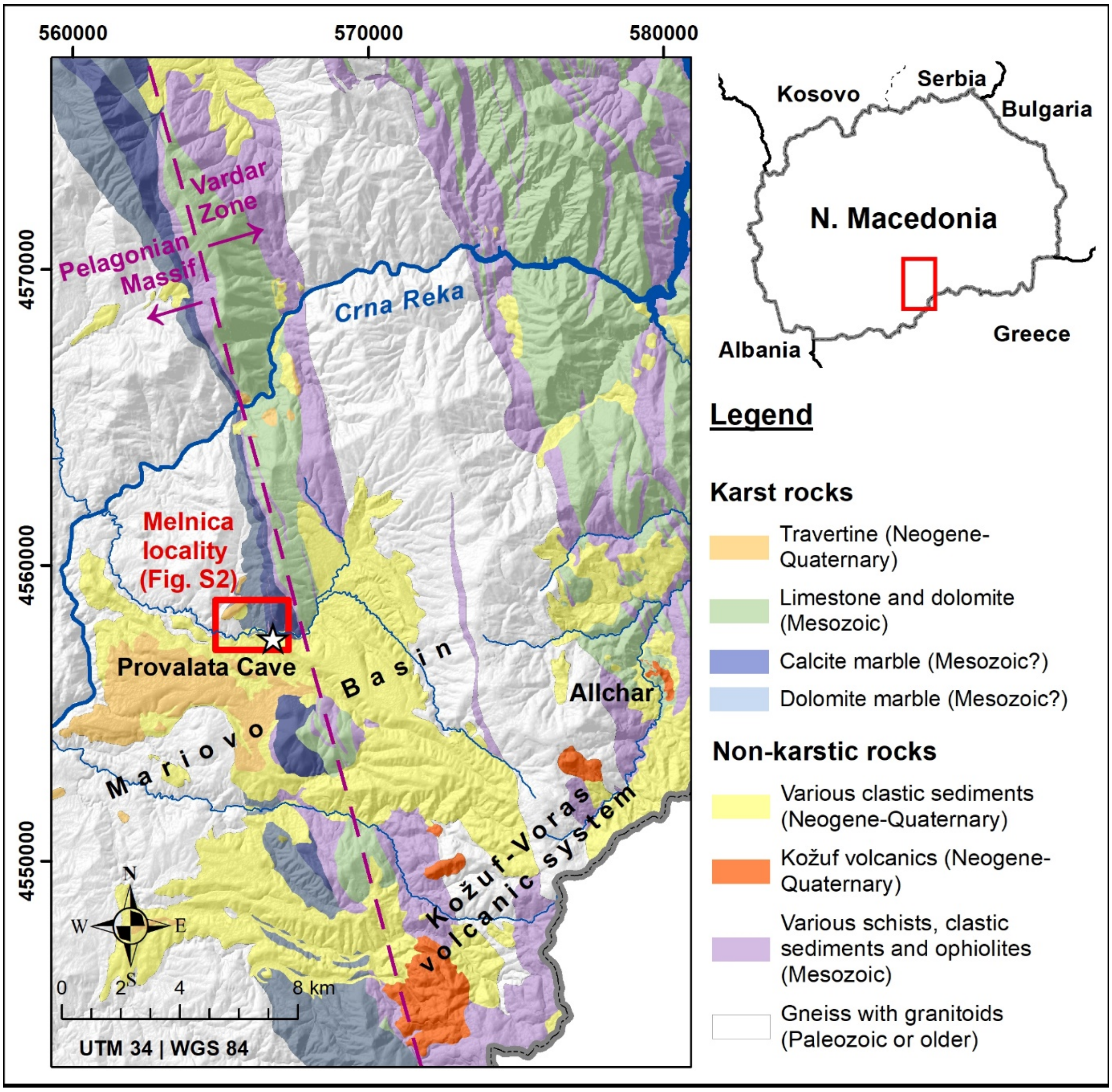

Figure S1. Simplified geological map of the area showing the NNW-SSE oriented nappe structures along border of the Pelagonian Massif and the Vardar Zone, with units of dolomite marble and calcite marble, and the location of Melnica locality (shown in detail in Fig. S2) and Provalata Cave. Allchar ore deposit and parts of Kožuf-Voras volcanic system are also indicated.

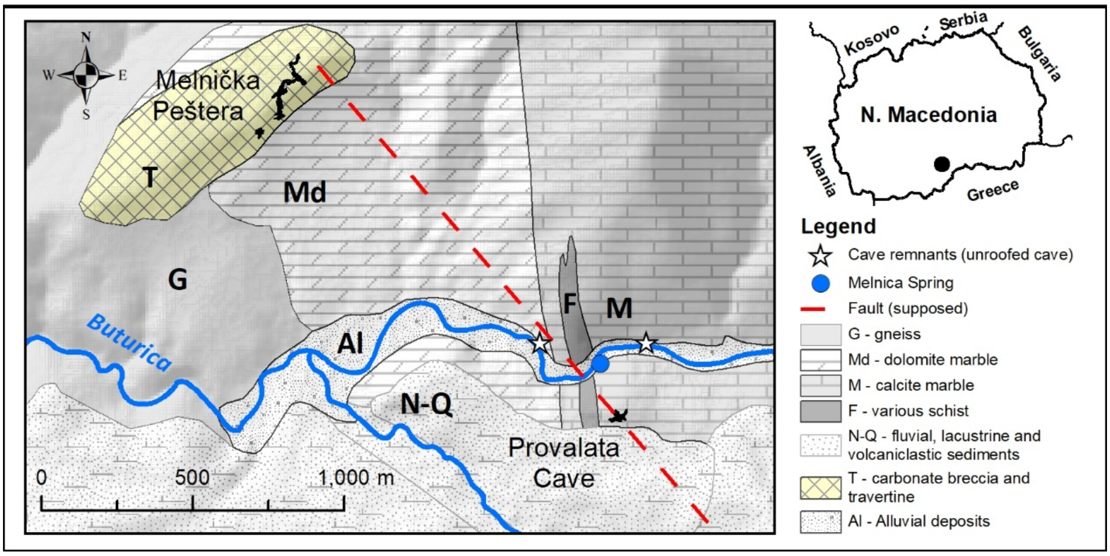

Figure S2. Geological setting of Provalata Cave in Melnica hypogene karst locality.

Sampling location and sample description

Samples were collected along the western and northern wall in the First Room of Provalata Cave (Figs.SS3, SS4). PR20 and PROV03 were cut from calcite coatings along the cave wall using circular saw with diamond blade. Two cores (C2 and C3) were drilled using 2.5 cm diameter corer through calcite coating and underlying marble bedrock, using the same approach as described in Spötl & Mattey (2012).

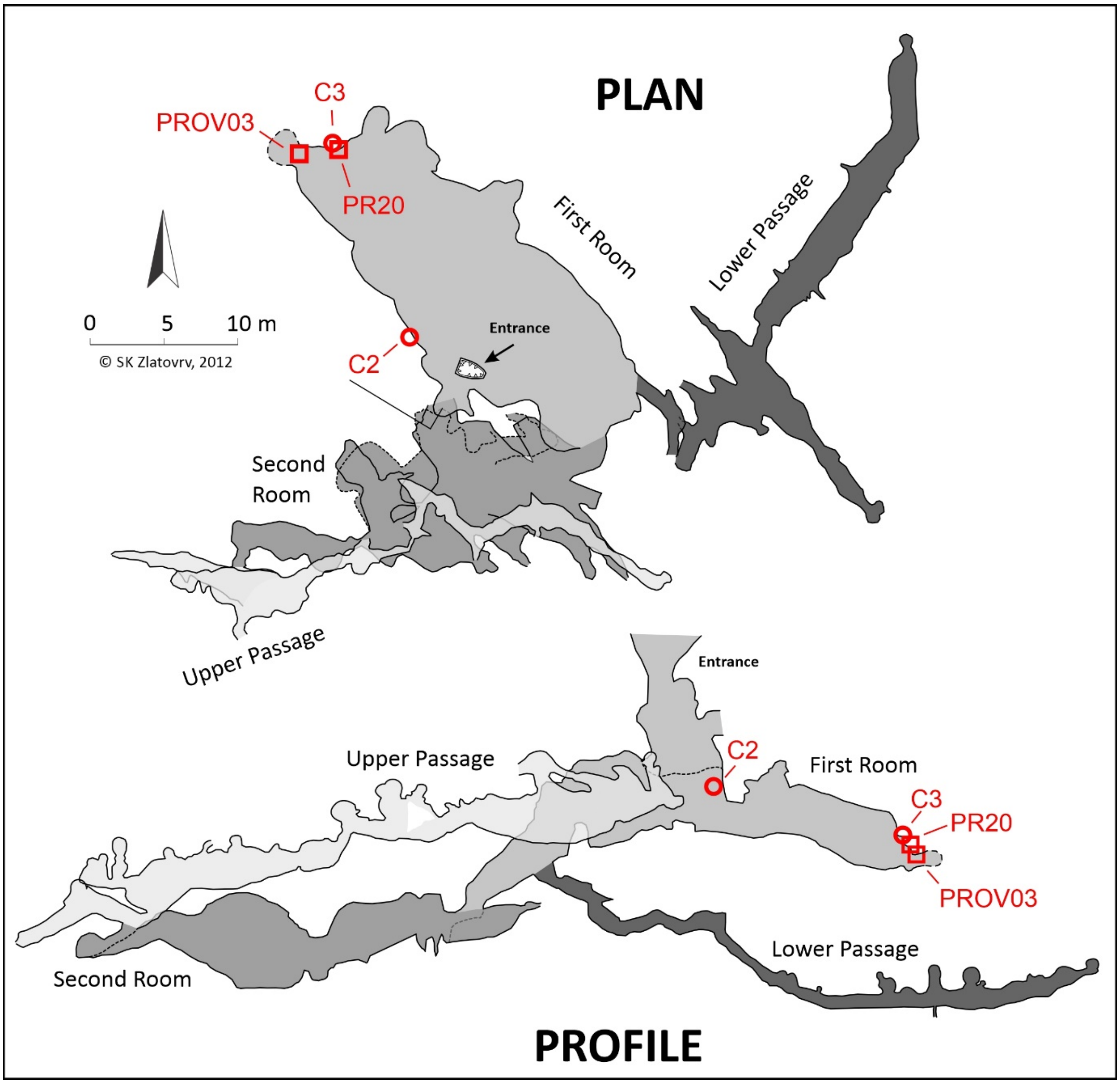

Figure S3. Location of studied Provalata Cave samples: circle – core sample; square – hand sample.

PR20 is ~ 20 mm thick calcite crust cut from the northern wall of the First Room. PROV03 is ~18 cm long cut through the marble bedrock and calcite coating from the norther side of the First Room, at the junction with a small niche. C2 is a ~30 cm long core drilled at the western wall in the First Room, close to the entrance shaft. It covers ~15 cm calcite coating and ~15 cm marble bedrock. C3 core is a small ~64 mm long core drilled next to where PR20 crust was sampled. The calcite coating in the core is thin (26 mm), as it was largely dissolved in the subsequent sulfuric acid speleogenetic phase.

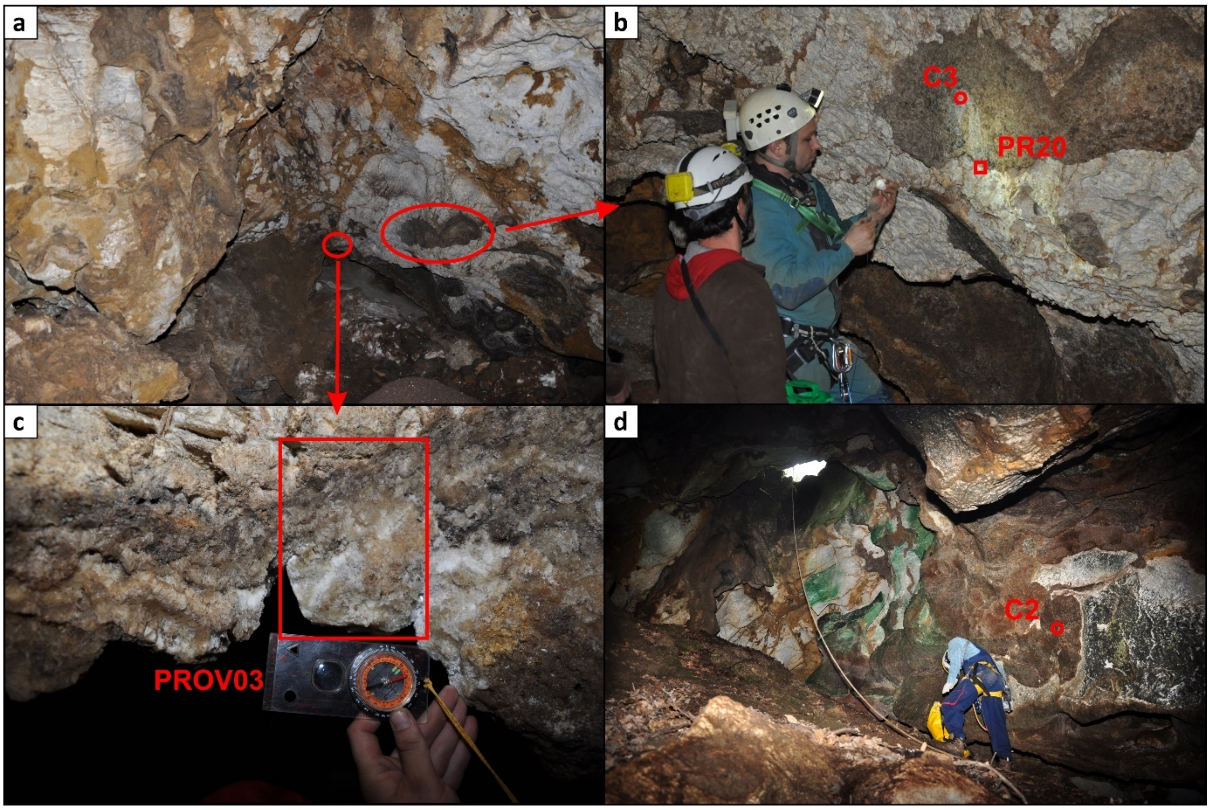

Figure S4. Location of core and hand samples from Provalata Cave. (**a**) View of the NW part of the First Room with approximate location of close-ups in (b) and (c). (**b**) Location of hand samples PR20 and core C3. (**c**) Location of hand sample PROV03. (**d**) View of the W part of the First Room, below the entrance shaft and location of core C2.

The collected samples were cut in half and grinded. Subsamples were drilled for carbonate stable isotope analysis by a hand drill along the sample length, perpendicular to the cave wall, and following the growth axis of the calcite coatings (Fig. S5). The marble section and the first 2 cm of the calcite coating at C2 were sampled at a resolution of 1-5 mm for conventional stable isotope analysis. Additionally, C2, and the other samples were drilled at a lower resolution for combined conventional and clumped stable isotope analysis. Three solid prisms were cut from the calcite coating in C2 at two locations for U-Pb and U-series analysis (Fig. S5).

Similarly, calcite marble bedrock samples collected along the NNW-SSE stripe (Fig.S1) were also hand-drilled for conventional and clumped stable isotope analysis.

Thin sections were prepared from samples PR20 and C2 for petrographic and fluid inclusion analysis.

For fluid inclusion stable isotope and noble gas analysis, subsamples were cut from PR20, C3 and PROV03 samples (Fig. S5).

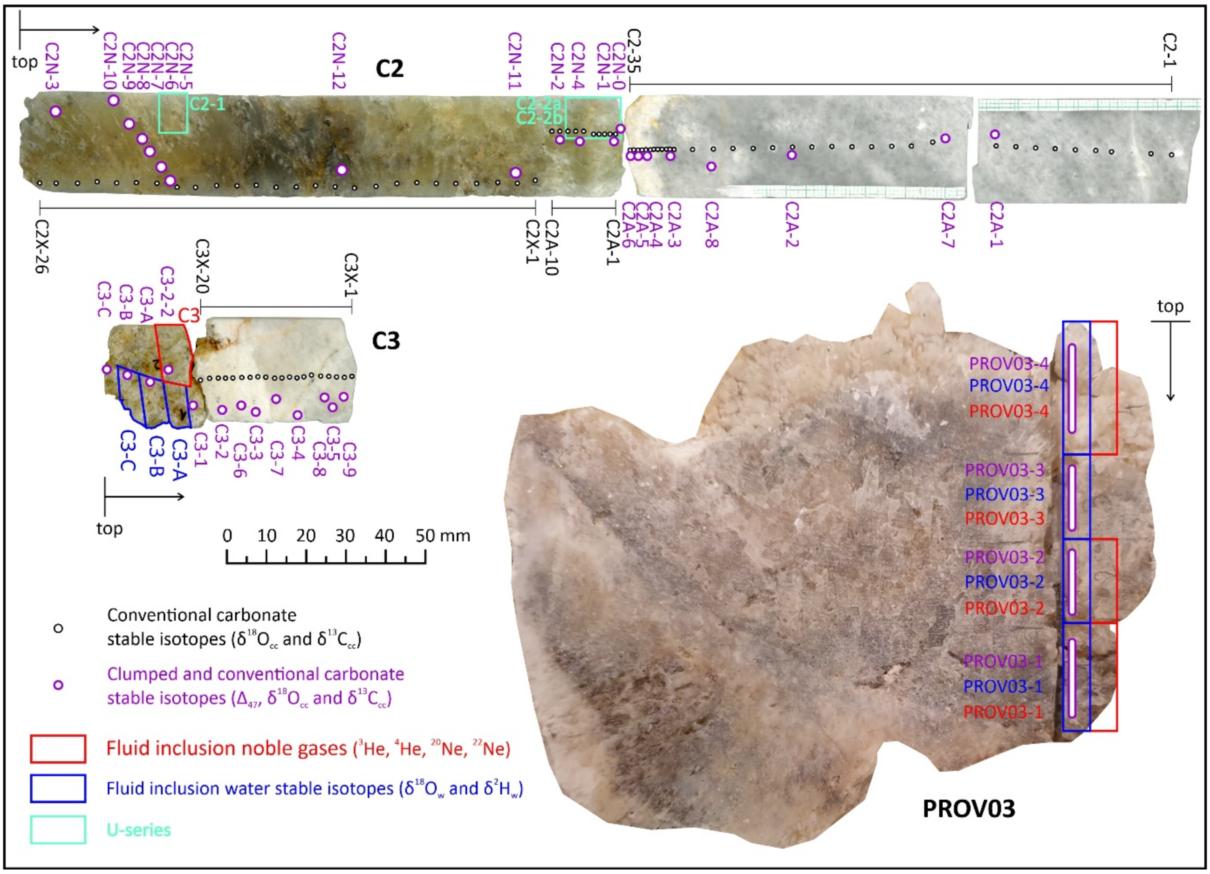

Figure S5. Studied samples from Provalata Cave and location of collected subsamples for various analyses.

Microscopic petrography

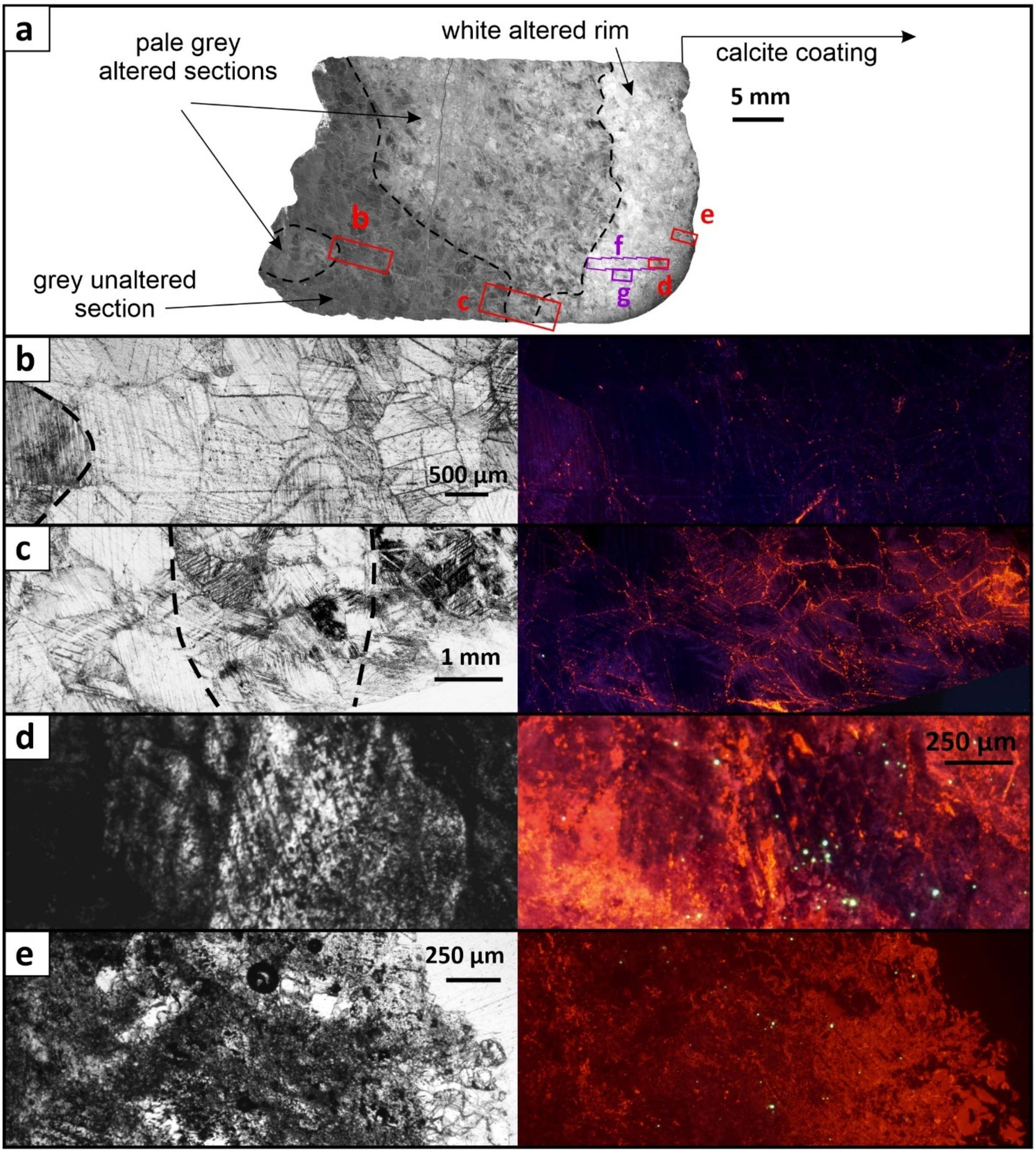

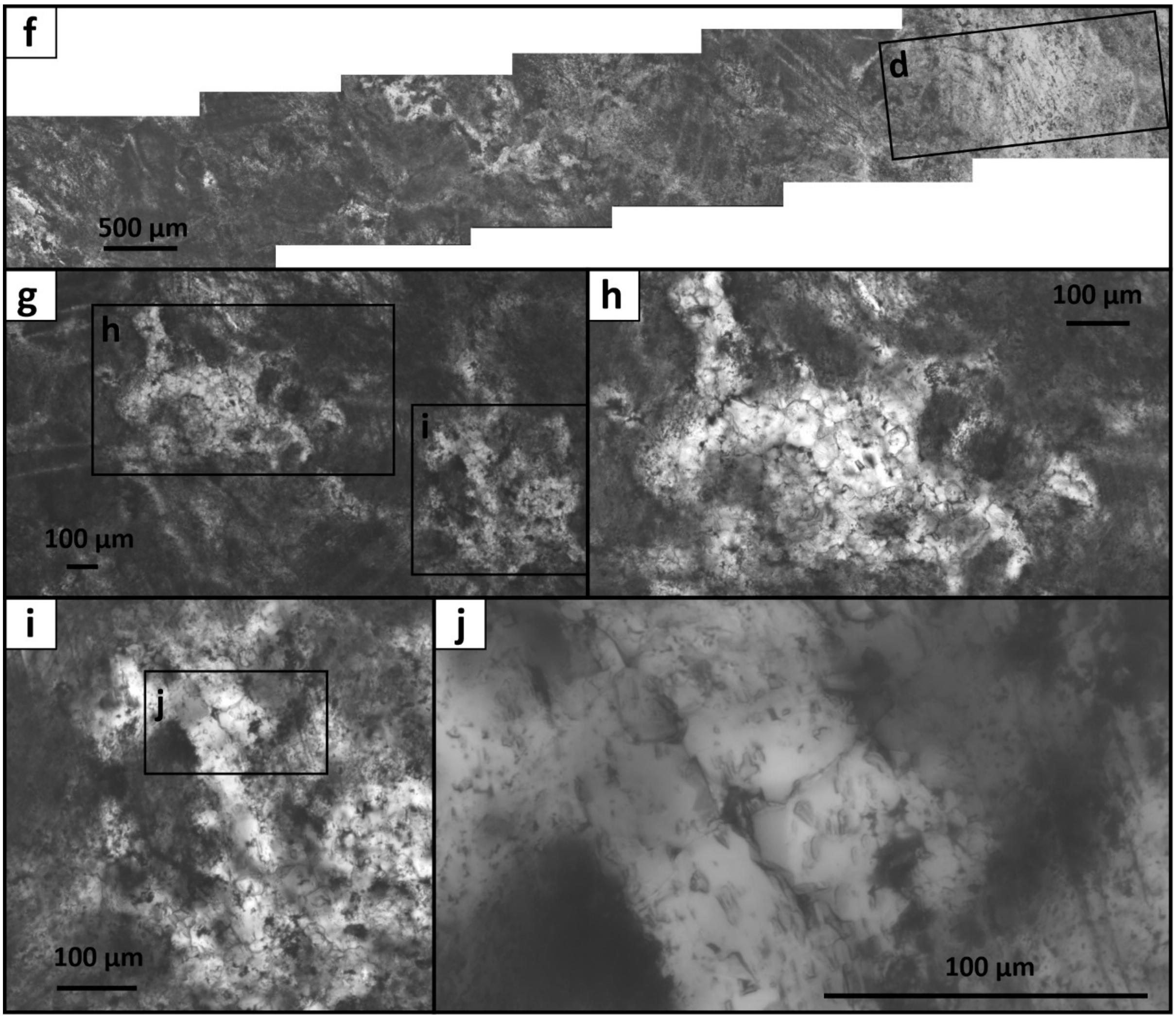

Figure S6. Photomicrographs of C2 core showing different sections of the calcite marble cave wall in plane-polarized transmitted light and cathodoluminescent light. (**a**) Overview of the thin section from the outer part of the core with location of the closer examined sections in b to g. (**b**) View of the unaltered section of the marble having large (mm size) crystals with dark blue luminescence. (**c**) A cross-section from the unaltered (left) through pale grey (middle) to white rim section, showing dark blue, violet and orange luminescence, respectively. (**d**) Part of the white rim, with remnants of a large calcite crystal with dark blue luminescence that changes outwards to violet, surrounded by smaller crystals with orange luminescence. (**e**) Outermost part of the alteration profile dominated by small crystals with orange luminescence, with larger crystals with orange luminescence visible towards the edge as part of the covering calcite coating. (**f-j**) Close-up view of the white altered rim section showing very small calcite crystals filling up pore spaces.

Fluid inclusion results

Provalata calcite samples are rich in fluid inclusions (Fig. S7). They are dominated by primary (intra-crystalline) inclusions, mostly as large size inclusions in palisade crystals (Fig. S7), but also as clouds of small 'thorn' shaped inclusions (Fig. S7), with different orientation of 'thorns' in a given inclusion cloud (Fig. S7) indicating formation of calcite crystals as an aggregate of several sub-crystals with different crystallographic orientation. Secondary (inter-crystalline) inclusions can also be present along crystal boundaries (Fig. S7), but their small size indicates that the sample water composition should be dominated by the water in the primary inclusions.

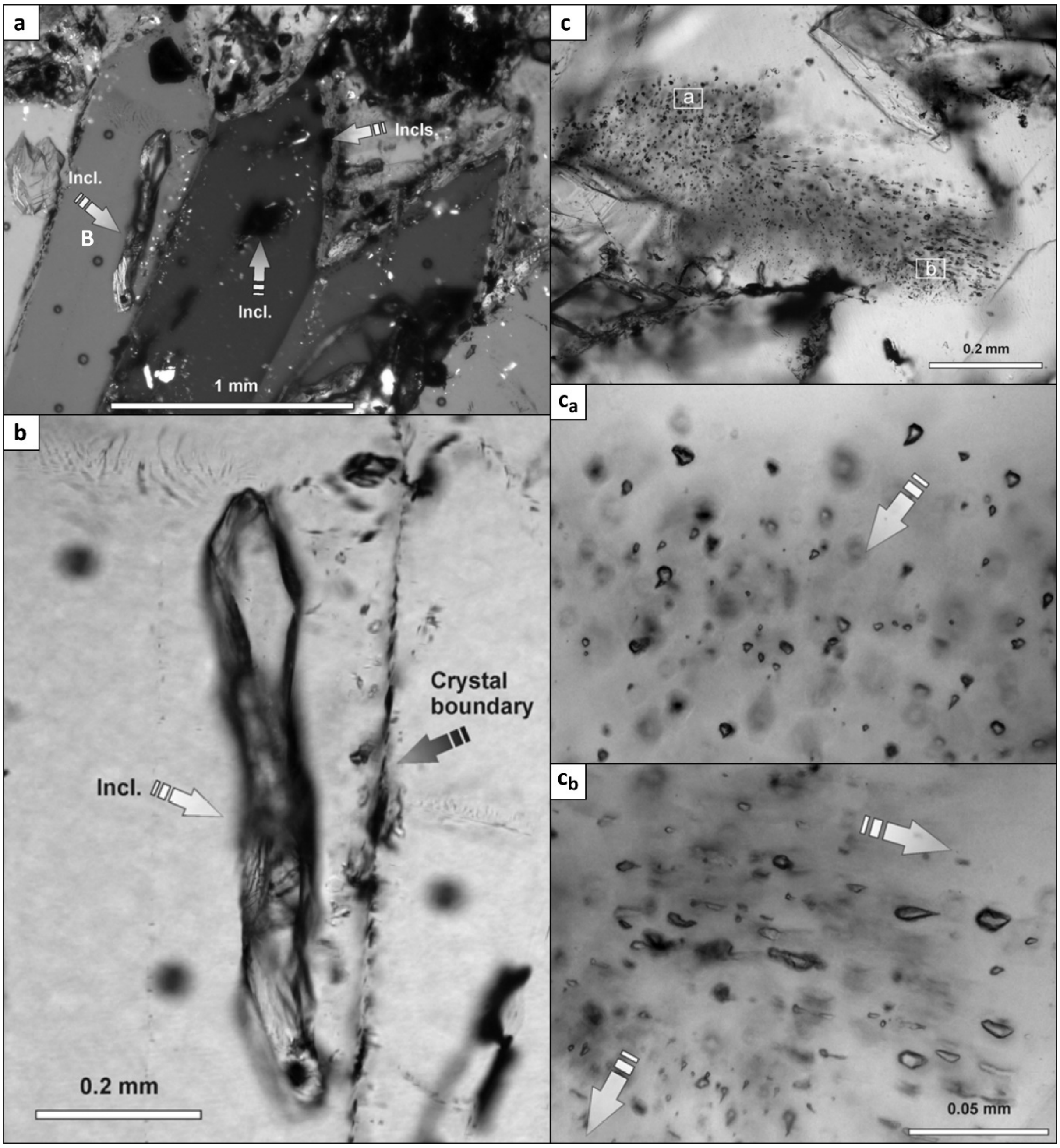

Figure S7. Fluid inclusion petrography of sample PR20. (**a**) Large intra-crystalline inclusions in palisade calcite crystals. (**b**) Closer view of the large intra-crystalline inclusion in **a**, and very small inter-crystalline inclusions along crystal boundary. (**c**) Cloud of small primary thorn-

shaped inclusions. (**c_a-c_b**) Close up views of areas (a) and (b) in panel (c), showing different orientation of the 'thorn' shaped inclusions.

Inclusion water was analyzed in 9 Provalata calcite samples (2 from PR20, 4 from PROV03 and 3 from C2). Sufficient water amount for stable isotope analyses was extracted only from 7 samples, with water content ranging between 0.48 and 4.38 µL/g. Inclusion water stable isotope composition indicates meteoric origin, with $\delta^{18}O_w$ values ranging between -13.9‰ and -11.6‰ and δ^2H_w values between -87.0‰ and -76.0‰ (Table S1).

Table S1. Water amount and isotopic composition of fluid inclusions from Melnica hydrothermal calcite. Inn - Institute of Geology, University of Innsbruck; CsFK - Institute for Geological and Geochemical Research, Budapest

Sample	Water content (µL/g)	δ^{18}O (‰ VSMOW)	δ^2H (‰ VSMOW)	Lab
PR20a	0.94	-12.0	-81.0	Inn
PR20b	0.48	-11.9	-83.6	Inn
PROV03-1	2.28	-11.9	-76.0	CsFK
PROV03-2	1.73	-13.1	-84.0	CsFK
PROV03-3	0.66	/	/	CsFK
PROV03-4	1.46	/	/	CsFK
C3-A	2.72	-13.9	-87.0	CsFK
C3-B	4.38	-12.4	-85.0	CsFK
C3-C	2.90	-11.6	-79.0	CsFK

Fluid inclusion noble gas composition was analyzed in 4 samples: one from C3 and three from PROV03. Helium concentrations are 8.7-40.6×10^{-9} ccSTP/g_{cc} and neon concentration 4.3-18.6×10^{-9} ccSTP/g_{cc}. Helium isotope ratios (R=^{3}He/^{4}He) ranged from 0.37 to 0.50 R/R_A (where R_A is the atmospheric ratio with value of 1.384×10^{-6}), with ^{4}He/^{20}Ne ratios from 1.5 to 2.4. Based on a three-end-member mixing model of atmospheric- (R/R_A = 1, ^{4}He/^{20}Ne = 0.318), crustal- (R/R_A = 0.02, ^{4}He/^{20}Ne = 1000) and mantle- (R/R_A = 8; ^{4}He/^{20}Ne = 1000) sourced helium and neon (Sano and Wakita 1985, Sano and Marty 1995, Graham 2002), crustal helium is the dominant component (76-84%), with mantle helium from 2-4% and atmospheric helium from 13-22% (Table S2).

Table S2. Noble gas composition of fluid inclusions from Melnica hydrothermal calcite. Atomki – Isotope Climatology and Environmental Research Centre, Institute for Nuclear Research, Debrecen

Sample	He (×10^{-9} ccSTP/g_{cc})	Ne (×10^{-9} ccSTP/g_{cc})	R/Ra	^{4}He/^{20}Ne	He-atm (%)	He-mantle (%)	He-crust (%)	Lab
C3	40.6	18.5	0.37	2.4	13	3	84	Atomki
PROV03-1	26.6	18.6	0.50	1.6	20	4	76	Atomki
PROV03-2	15.6	11.8	0.39	1.5	22	2	76	Atomki
PROV03-4	8.7	4.3	0.32	2.3	14	2	84	Atomki

S5. Uranium-series results

Table S3. U-series results for calcite coating in core C2. All uncertainties are absolute 2σ, and all ratios are activity.

Sample	^{238}U concentration (ppb)		^{232}Th concentration (ppb)		$^{230}Th/^{232}Th$		$^{230}Th/^{238}U$		$\delta^{234}U$ (‰)	
C2-1	77.01	± 0.06	826	± 18	288.38	± 6.41	1.012	± 0.002	10	± 1
C2-2a	32.98	± 0.05	10115	± 28	10.24	± 0.06	1.028	± 0.006	22	± 2
C2-2b	29.87	± 0.18	3913	± 31	23.96	± 0.27	1.027	± 0.010	8	± 4

Modeling of carbon isotope composition in secondary calcite minerals

For the modeling of the $\delta^{13}C_{cc}$ values in terms of calcite precipitation due to change in temperature with or without CO_2 degassing, we have used the equation of Zheng (1990) with a modification, that instead of considering two extreme cases of HCO_3-dominant or H_2CO_3-dominant fluid, for which only fractionation of calcite-HCO_3 or calcite-H_2CO_3 is used, respectively, we select a set of fractions of H_2CO_3 in the DIC of the fluid (where DIC = fH_2CO_3 + (1-f) × HCO_3), that itself reflects fluid pH at a given T. Thus, for a selected $\delta^{13}C_{DIC}$ (-1 to +4 ‰), range of temperatures (5-40 °C) and fH_2CO_3 (0.3-0.7), first we calculate the $\delta^{13}C$ of the CO_2 in equilibrium with the fluid ($\delta^{13}C_{CO2}$) from the relationship:

$$\delta^{13}C_{DIC} = fH_2CO_3 \times (\delta^{13}C_{CO2} + 1000\ln\alpha_{H2CO3\text{-}CO2}) + (1- fH_2CO_3) \times (\delta^{13}C_{CO2} + 1000\ln\alpha_{HCO3\text{-}CO2}) \quad (1)$$

and then we calculate $\delta^{13}C_{cc}$ values using the Rayleigh model equation of Zheng (1990), in the form of:

$$\delta^{13}C_{cc} = \delta^{13}C_{CO2} + [1 + \ln \times (1\text{-}2X_c)] \times 1000\ln\alpha_{cc\text{-}CO2} \quad (2)$$

where X_c is the mole fraction of carbon in the degassed CO_2, assuming that chemically the mol fraction of carbon lost due to CO_2 degassing is identical to the one lost through calcite precipitation, and $1000\ln\alpha_{H2CO3\text{-}CO2}$, $1000\ln\alpha_{HCO3\text{-}CO2}$ and $1000\ln\alpha_{cc\text{-}CO2}$ are the temperature dependent carbon fractionation factors of H_2CO_3, HCO_3 and calcite with CO_2, respectively (Mook 2000).

Modeled curves are shown for 0.1 mol fraction of carbon in degassed CO_2 (Fig. S8) and no CO_2 degassing (Fig. S9).

The modeled curves show characteristic slopes of T-$\delta^{13}C_{cc}$ for a range of fH_2CO_3 (Figs. S8, S9), having positive slope at low fH_2CO_3 (higher pH) and slightly negative slope at higher fH_2CO_3 (lower pH). For a given fH_2CO_3, the intercept is primarily controlled by the $\delta^{13}C_{DIC}$, but also by the degassing, with higher X_c value showing lower intercept. Negative slope is maintained only for conditions of higher fH_2CO_3 and no or low degassing (>0.5 fH_2CO_3 at 0 X_c, >0.6 fH_2CO_3 at 0.1 X_c and >0.7 fH_2CO_3 at 0.2 X_c).

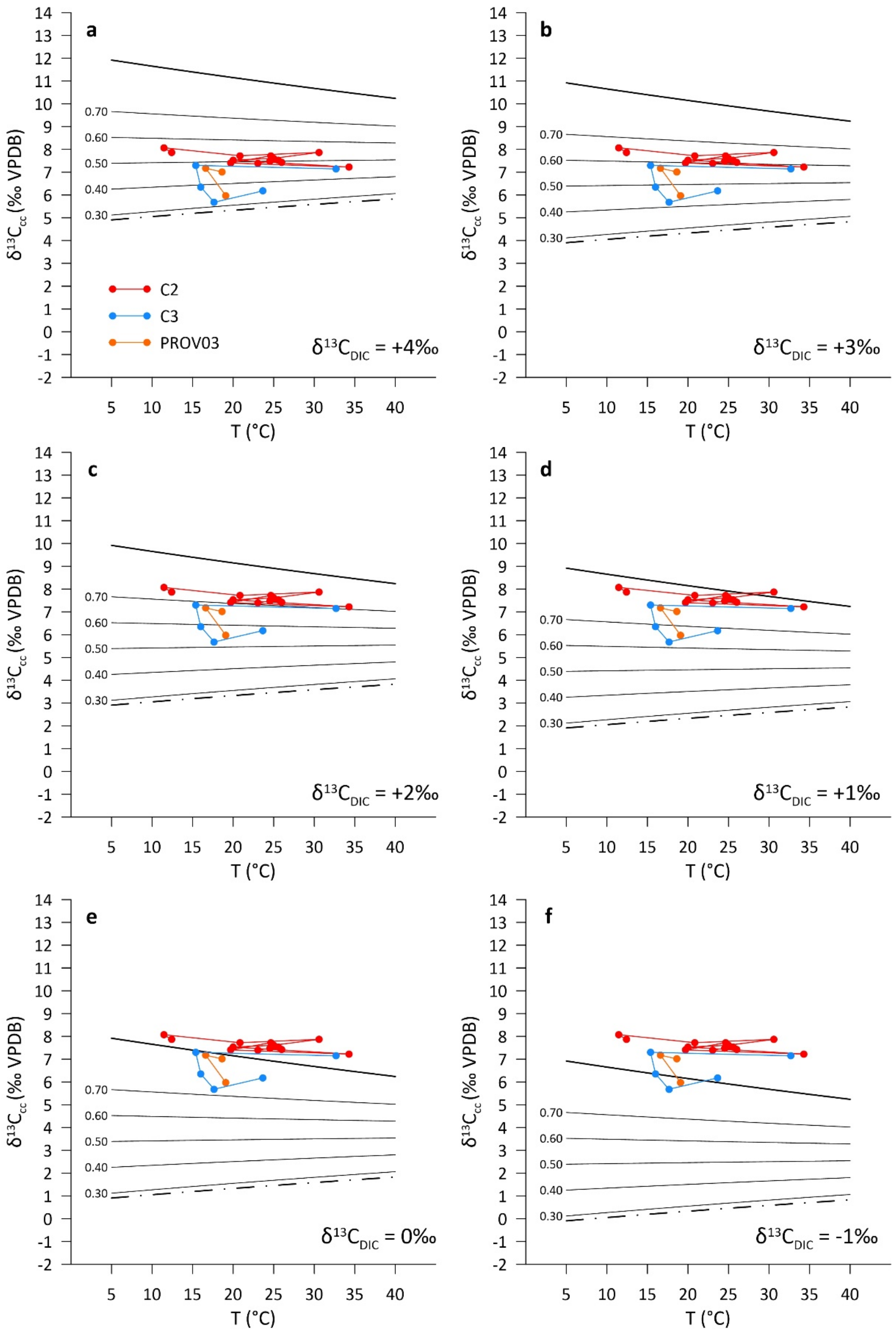

Figure S8. Modeling of change in $\delta^{13}C_{cc}$ with change in temperature for $f H_2CO_3$ of 0.3 to 0.7, and a $\delta^{13}C_{DIC}$ of -1‰ to +4‰ (**a-f**), for an X_c of 0.1 (i.e., 0.1 mole fraction of carbon in the degassed CO_2). Also shown are curves for H_2CO_3-dominant (thick full line) and HCO_3-dominant fluid (thick dash-dot line), calculated using the original equation of Zheng (1990).

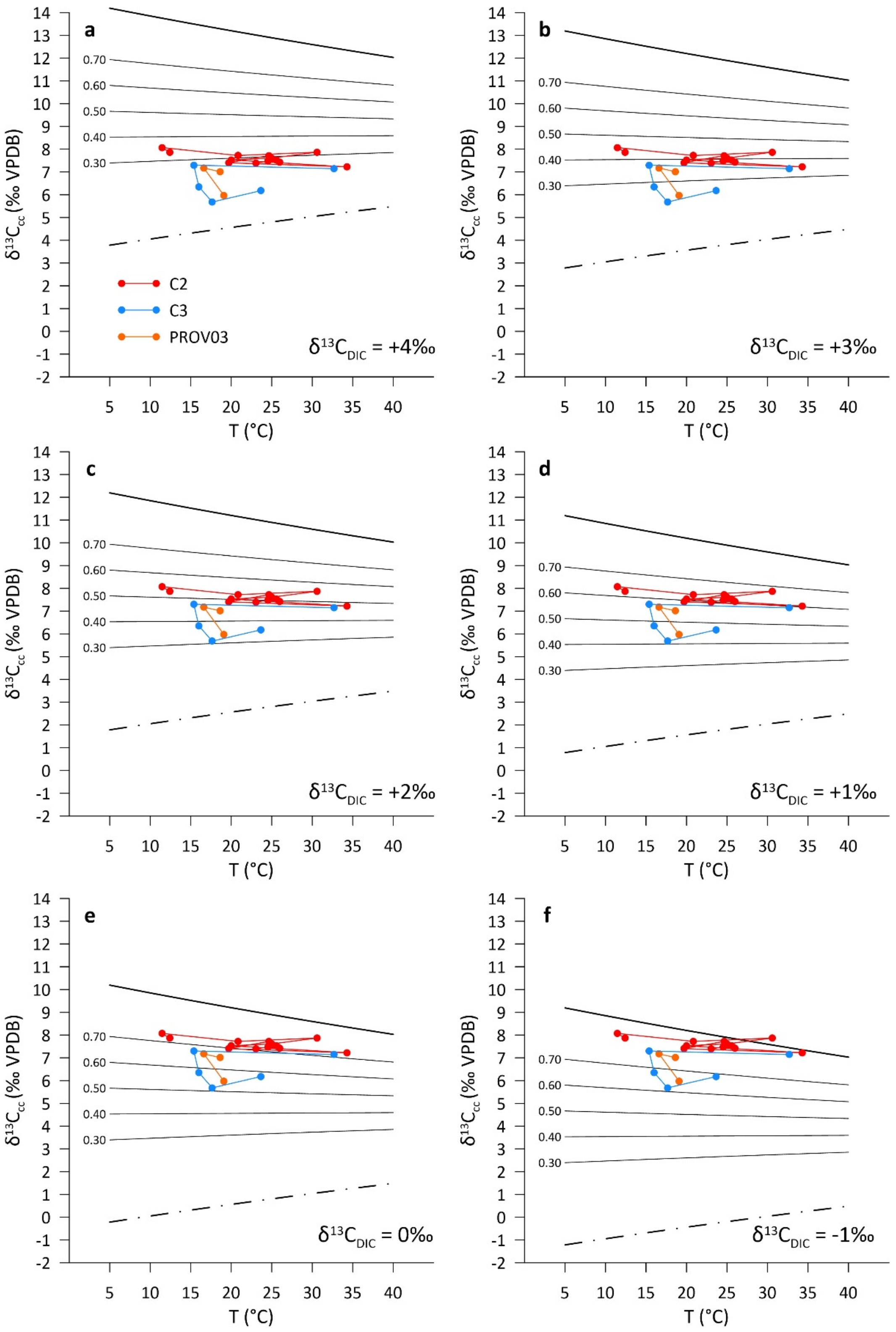

Figure S9. Modeling of change in $\delta^{13}C_{cc}$ with change in temperature for fH_2CO_3 of 0.3 to 0.7, and a $\delta^{13}C_{DIC}$ of -1‰ to +4‰ (**a-f**), for an X_c of 0 (no CO_2 degassing). Also shown are curves for H_2CO_3-dominant (thick full line) and HCO_3-dominant fluid (thick dash-dot line), calculated using the original equation of Zheng (1990).

To gain insight into the composition of the DIC at the onset of calcite deposition in C2 core ($\delta^{13}C_{cc}$ value of +7.9‰ and apparent temperature of 12°C), we model the $\delta^{13}C_{DIC}$ value of a calcite saturated fluid ($SI_{cc} \geq 0$) at a range of pH values and temperature of 12°C, so that the calcite precipitating from it under isotopic equilibrium conditions, has $\delta^{13}C_{cc}$ value of +7.9‰. We first calculate the fractions of H_2CO_3 and HCO_3 in PHREEQC Version 3 software (Parkhurst and Appelo 2013) for a range of pH values (6 to 7.6), with temperature set at 12°C, and calcium and DIC concentrations determined by forcing charge balance to the solution at SI_{cc} of 0.01.

We determined the $\delta^{13}C_{CO2}$ value for the CO_2 in equilibrium with calcite with $\delta^{13}C_{cc}$ value of +7.9‰ from:

$\delta^{13}C_{CO2} = \delta^{13}C_{cc} - 1000\ln\alpha_{cc\text{-}CO2}$ (3)

and then we calculate the $\delta^{13}C_{DIC}$ value using equation (1).

Using the PHREEQC modeled Ca^{2+} and DIC concentrations, we can separate the fractions of carbon in the DIC (C_{DIC}) derived from dissolution of $CaCO_3$ (C_{rock}) and from external CO_2 (C_{ext}):

$C_{DIC} = f \times C_{rock} + (1\text{-}f) \times C_{ext}$ (4)

The isotopic composition of the external CO_2 i.e., CO_2 not accounted for by dissolution of carbonate rocks ($\delta^{13}C_{ext}$) can be determined from the following equation (Chiodini et al. 2000):

$(\delta^{13}C_{ext} \times f_{Cext}) = (\delta^{13}C_{DIC}) - (\delta^{13}C_{rock} \times f_{Crock})$ (5)

It should be noted that the outlined approach considers only pure $CaCO_3$ dissolution, i.e., the rock component is represented solely by the Ca^{2+} ion concentration. If Mg^{2+} is also introduced, e.g., by dissolution of dolomite, calcite saturation can be reached at lower Ca^{2+} concentrations, but there will be no significant change in the $\delta^{13}C_{DIC}$, and if the dolomite has similar $\delta^{13}C_{rock}$, there will be no significant change in the $\delta^{13}C_{ext}$ as well. To demonstrate, we do the same water chemistry calculation in PHREEQC, but we include Mg^{2+} concentration that is half the one of Ca^{2+} calculated previously. The DIC and Ca^{2+} are constrained in the same way by forcing charge balance and SI_{cc} of 0.01. The final Mg/Ca ratio of the modeled compositions is 0.6.

Modeled values using $\delta^{13}C_{rock}$ of +2.5‰, as found for the calcite marble formation, and SI_{cc} of 0.01 are given in Table S4 and curves are shown in Fig. S10.

Table S4. Modeled chemical and isotopic composition of a fluid for a range of pH values at a set temperature of 12°C and SI_{cc} of 0.01 so that calcite can precipitate in isotopic equilibrium with a $\delta^{13}C_{cc}$ value of +7.9‰.

Dissolved minerals	pH	fH_2CO_3	$fHCO_3$	DIC	Ca	Mg	pCO_2 (atm)	fC_{rock}	fC_{ext}	$\delta^{13}C_{DIC}$ (‰)	$\delta^{13}C_{ext}$ (‰)
				mmol/L							
calcite	7.6	0.06	0.94	3.0	1.4		0.004	0.48	0.52	6.9	10.9
	7.4	0.09	0.91	4.0	1.9		0.007	0.46	0.54	6.6	10.1
	7.2	0.14	0.86	5.5	2.4		0.015	0.44	0.56	6.1	8.9
	7.0	0.20	0.80	7.6	3.1		0.031	0.41	0.59	5.4	7.5
	6.8	0.28	0.72	11.0	4.1		0.062	0.37	0.63	4.6	5.8
	6.6	0.38	0.62	16.6	5.3		0.127	0.32	0.68	3.5	4.0
	6.4	0.49	0.51	26.4	7.0		0.260	0.26	0.74	2.4	2.3
	6.2	0.60	0.40	44.2	9.2		0.532	0.21	0.79	1.2	0.9
	6.0	0.70	0.30	77.7	12.2		1.093	0.16	0.84	0.2	-0.2
calcite + dolomite (Mg/Ca = 0.6)	7.6	0.06	0.94	3.9	1.2	0.7	0.005	0.48	0.52	6.9	11.0
	7.4	0.09	0.91	5.2	1.5	0.9	0.010	0.46	0.54	6.6	10.1
	7.2	0.14	0.86	7.1	2.0	1.2	0.020	0.44	0.56	6.1	9.0
	7.0	0.20	0.80	10.0	2.5	1.6	0.040	0.41	0.59	5.5	7.5
	6.8	0.28	0.72	14.4	3.3	2.0	0.081	0.37	0.63	4.6	5.8
	6.6	0.38	0.62	21.7	4.4	2.6	0.165	0.32	0.68	3.6	4.1
	6.4	0.49	0.51	34.5	5.8	3.5	0.337	0.27	0.73	2.4	2.4
	6.2	0.60	0.40	57.7	7.7	4.6	0.691	0.21	0.79	1.3	1.0
	6.0	0.70	0.30	101.3	10.3	6.1	1.422	0.16	0.84	0.2	-0.2

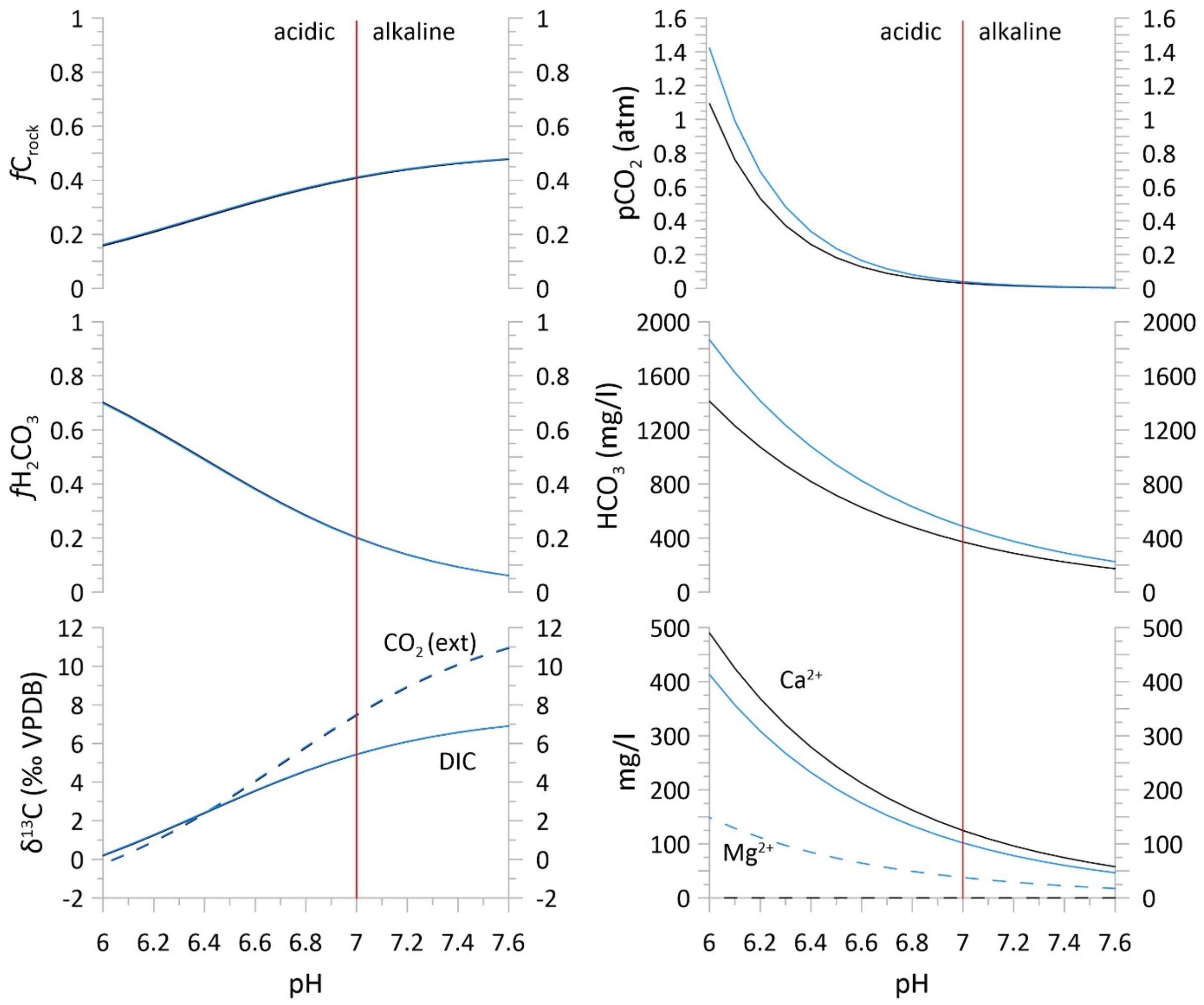

Figure S10. Modeled chemical and isotopic composition of a calcite saturated fluid (SI_{cc} set at 0.01) with temperature of 12°C for a range of pH values, from which calcite can precipitate in isotopic equilibrium with $\delta^{13}C_{cc}$ value of +7.9‰. Black lines are show compositions where only calcite dissolution is considered, and blue lines where dolomite dissolution is also included, for which the final modeled compositions have Mg/Ca of 0.6.

Table S5. Supplementary dataset

No.	Location	Sample type	Sample	Subsample	Carbonate stable isotopes											Water stable isotopes (calculated or measurements on fluid inclusion)		Fluid inclusion noble gas data								
					Conventional				Clumped																	
					$\delta^{18}O_{cc}$	±1SD	$\delta^{13}C_{cc}$	±1SD	Δ_{47}	±1SD	±1SE	±95% CL	T	±1SE	Repli-cates	$\delta^{18}O_w$	±1SD	R/R_a	±1SE	$^4He/^{20}Ne$	±1SE	Atmospheric Helium	Mantle Helium	Crustal Helium	Distance from top	comment
					‰ VPDB				‰ I-CDES90				°C		n	‰ VSMOW		%		%		%			mm	
1	Provalata Cave	calcite marble	C2	C2-1	-5.53	0.10	2.45	0.08																	289.5	
2	Provalata Cave	calcite marble	C2	C2-2	-4.79	0.10	2.54	0.08																	284.3	
3	Provalata Cave	calcite marble	C2	C2-4	-6.20	0.10	2.76	0.08																	274.2	
4	Provalata Cave	calcite marble	C2	C2-5	-5.02	0.10	2.58	0.08																	270.3	
5	Provalata Cave	calcite marble	C2	C2-6	-5.48	0.10	2.61	0.08																	265.1	
6	Provalata Cave	calcite marble	C2	C2-7	-4.89	0.10	2.68	0.08																	260.2	
7	Provalata Cave	calcite marble	C2	C2-8	-7.23	0.10	2.51	0.08																	255.3	
8	Provalata Cave	calcite marble	C2	C2-9	-6.97	0.10	2.51	0.08																	250.3	
9	Provalata Cave	calcite marble	C2	C2-10	-8.84	0.10	2.56	0.08																	245.4	
10	Provalata Cave	calcite marble	C2	C2A-1	-7.07	0.19	2.72	0.05	0.375	0.038	0.010	0.021	146	9	15	10.9	1.1								245.1	
11	Provalata Cave	calcite marble	C2	C2A-7	-8.18	0.07	2.69	0.01	0.417	0.029	0.008	0.018	112	6	12	5.9	0.8								232.6	
12	Provalata Cave	calcite marble	C2	C2-11	-6.26	0.10	2.55	0.08																	229.3	
13	Provalata Cave	calcite marble	C2	C2-12	-5.03	0.10	2.60	0.08																	224.0	
14	Provalata Cave	calcite marble	C2	C2-13	-5.86	0.10	2.63	0.08																	219.1	
15	Provalata Cave	calcite marble	C2	C2-14	-4.34	0.10	2.56	0.08																	213.9	
16	Provalata Cave	calcite marble	C2	C2-15	-8.13	0.10	2.54	0.08																	208.9	
17	Provalata Cave	calcite marble	C2	C2-16	-4.32	0.10	2.42	0.08																	203.8	
18	Provalata Cave	calcite marble	C2	C2-17	-3.43	0.10	2.50	0.08																	198.9	
19	Provalata Cave	calcite marble	C2	C2-18	-3.39	0.10	2.54	0.08																	193.9	
20	Provalata Cave	calcite marble	C2	C2A-2	-3.51	0.03	2.64	0.01	0.330	0.031	0.008	0.017	196	10	16	18.9	0.9								193.9	
21	Provalata Cave	calcite marble	C2	C2-19	-3.41	0.10	2.45	0.08																	188.9	
22	Provalata Cave	calcite marble	C2	C2-20	-3.97	0.10	2.55	0.08																	184.0	
23	Provalata Cave	calcite marble	C2	C2-21	-4.54	0.10	2.45	0.08																	179.0	
24	Provalata Cave	calcite marble	C2	C2-22	-7.71	0.10	2.39	0.08																	174.0	
25	Provalata Cave	calcite marble	C2	C2A-8	-6.22	0.12	2.69	0.04	0.377	0.040	0.011	0.025	144	11	12	11.5	1.2								173.7	
26	Provalata Cave	calcite marble	C2	C2-23	-4.39	0.10	2.53	0.08																	168.8	
27	Provalata Cave	calcite marble	C2	C2-24	-4.11	0.10	2.53	0.08																	164.1	
28	Provalata Cave	calcite marble	C2	C2-25	-3.80	0.10	2.50	0.08																	163.2	
29	Provalata Cave	calcite marble	C2	C2A-3	-3.55	0.04	2.67	0.01	0.342	0.028	0.007	0.015	181	9	16	17.7	0.8								162.4	
30	Provalata Cave	calcite marble	C2	C2-26	-4.38	0.10	2.53	0.08																	162.1	
31	Provalata Cave	calcite marble	C2	C2-27	-3.90	0.10	2.51	0.08																	161.1	
32	Provalata Cave	calcite marble	C2	C2-28	-4.83	0.10	2.63	0.08																	160.2	
33	Provalata Cave	calcite marble	C2	C2-29	-7.37	0.10	3.23	0.08																	159.2	
34	Provalata Cave	calcite marble	C2	C2-30	-8.78	0.10	4.15	0.08																	158.3	
35	Provalata Cave	calcite marble	C2	C2A-4	-9.88	0.03	5.44	0.01	0.526	0.031	0.008	0.017	50	3	16	-4.5	0.6								157.5	
36	Provalata Cave	calcite marble	C2	C2-31	-7.77	0.10	4.46	0.08																	157.0	
37	Provalata Cave	calcite marble	C2	C2-32	-8.59	0.10	4.50	0.08																	156.2	
38	Provalata Cave	calcite marble	C2	C2A-5	-8.62	0.10	4.54	0.08	0.504	0.055	0.014	0.031	60	7	15	-1.5	1.2								155.3	
39	Provalata Cave	calcite marble	C2	C2-33	-9.06	0.10	4.69	0.08																	155.2	
40	Provalata Cave	calcite marble	C2	C2-34	-9.53	0.10	5.69	0.08																	154.1	
41	Provalata Cave	calcite marble	C2	C2A-6	-9.67	0.07	5.80	0.03	0.534	0.027	0.007	0.014	47	3	16	-4.8	0.6								153.3	
42	Provalata Cave	calcite marble	C2	C2-35	-10.16	0.10	5.97	0.08																	153.2	
43	Provalata Cave	calcite coating	C2	C2N-0	-11.33	0.07	7.87	0.06	0.631	0.039	0.010	0.022	12	3	14	-13.2	0.7								150.5	
44	Provalata Cave	calcite coating	C2	C2A1	-11.02	0.10	7.96	0.08																	149.5	
45	Provalata Cave	calcite coating	C2	C2N-1	-11.25	0.11	8.07	0.07	0.635	0.077	0.022	0.049	11	7	12	-13.3	1.5								148.8	
46	Provalata Cave	calcite coating	C2	C2A2	-11.12	0.10	7.87	0.08																	148.2	
47	Provalata Cave	calcite coating	C2	C2A3	-11.12	0.10	7.72	0.08																	146.8	
48	Provalata Cave	calcite coating	C2	C2A4	-11.12	0.10	7.82	0.08																	145.4	
49	Provalata Cave	calcite coating	C2	C2A5	-11.22	0.10	7.64	0.08																	144.0	
50	Provalata Cave	calcite coating	C2	C2A6	-11.42	0.10	7.50	0.08																	141.5	
51	Provalata Cave	calcite coating	C2	C2N-4	-11.57	0.07	7.72	0.05	0.604	0.027	0.008	0.017	21	3	12	-11.7	0.6								140.7	
52	Provalata Cave	calcite coating	C2	C2A7	-11.42	0.10	7.47	0.08																	139.5	
53	Provalata Cave	calcite coating	C2	C2A8	-11.32	0.10	7.64	0.08																	137.5	
54	Provalata Cave	calcite coating	C2	C2A9	-11.22	0.10	7.85	0.08																	135.5	
55	Provalata Cave	calcite coating	C2	C2N-2	-11.43	0.09	7.87	0.04	0.576	0.049	0.014	0.031	31	5	12	-9.6	1.0								135.5	
56	Provalata Cave	calcite coating	C2	C2A10	-11.32	0.10	7.73	0.08																	133.5	
57	Provalata Cave	calcite coating	C2	C2X-01	-11.82	0.10	6.78	0.08																	129.3	
58	Provalata Cave	calcite coating	C2	C2X-02	-11.85	0.10	7.58	0.08																	124.8	
59	Provalata Cave	calcite coating	C2	C2N-11	-12.01	0.07	7.61	0.04	0.59	0.04	0.01	0.02	25	4	12	-11.20	0.80								124.4	
60	Provalata Cave	calcite coating	C2	C2X-03	-11.96	0.10	7.55	0.08																	119.9	
61	Provalata Cave	calcite coating	C2	C2X-04	-11.86	0.10	7.56	0.08																	114.5	
62	Provalata Cave	calcite coating	C2	C2X-05	-12.15	0.10	7.30	0.08																	109.3	
63	Provalata Cave	calcite coating	C2	C2X-08	-12.11	0.10	7.17	0.08																	104.5	
64	Provalata Cave	calcite coating	C2	C2X-06	-11.90	0.10	7.41	0.08																	104.4	
65	Provalata Cave	calcite coating	C2	C2X-07	-11.91	0.10	7.42	0.08																	99.5	
66	Provalata Cave	calcite coating	C2	C2X-09	-11.83	0.10	7.60	0.08																	89.2	
67	Provalata Cave	calcite coating	C2	C2X-10	-11.76	0.10	7.39	0.08																	83.8	
68	Provalata Cave	calcite coating	C2	C2N-12	-12.03	0.03	7.53	0.02	0.61	0.03	0.01	0.02	20	3	11	-12.40	0.60								80.6	
69	Provalata Cave	calcite coating	C2	C2X-11	-11.97	0.10	7.41	0.08																	78.9	
70	Provalata Cave	calcite coating	C2	C2X-12	-11.95	0.10	7.40	0.08																	74	
71	Provalata Cave	calcite coating	C2	C2X-13	-12.13	0.10	7.28	0.08																	69.1	
72	Provalata Cave	calcite coating	C2	C2X-14	-12.02	0.10	7.39	0.08																	64.1	
73	Provalata Cave	calcite coating	C2	C2X-15	-11.53	0.10	6.85	0.08																	59	
74	Provalata Cave	calcite coating	C2	C2X-16	-11.93	0.10	7.34	0.08																	54.2	
75	Provalata Cave	calcite coating	C2	C2X-17	-12.06	0.10	7.13	0.08																	49.1	
76	Provalata Cave	calcite coating	C2	C2X-18	-12.11	0.10	7.24	0.08																	43.7	
77	Provalata Cave	calcite coating	C2	C2X-19	-12.13	0.10	7.08	0.08																	39.1	
78	Provalata Cave	calcite coating	C2	C2N-5	-12.13	0.19	7.22	0.07	0.566	0.029	0.008	0.018	34	3	12	-9.6	0.8								37.9	
79	Provalata Cave	calcite coating	C2	C2N-6	-12.09	0.06	7.41	0.02	0.608	0.035	0.010	0.022	20	3	12	-12.4	0.7								35.3	
80	Provalata Cave	calcite coating	C2	C2X-20	-11.84	0.10	7.21	0.08																	34	
81	Provalata Cave	calcite coating	C2	C2N-7	-11.86	0.05	7.72	0.02	0.593	0.021	0.006	0.014	25	2	11	-11.2	0.5								32.2	
82	Provalata Cave	calcite coating	C2	C2N-8	-11.95	0.07	7.53	0.03	0.590	0.036	0.010	0.022	26	3	13	-11.1	0.7								29.9	
83	Provalata Cave	calcite coating	C2	C2X-21	-11.93	0.10	7.44	0.08																	28.9	
84	Provalata Cave	calcite coating	C2	C2N-9	-12.24	0.09	7.43	0.03	0.589	0.051	0.013	0.027	26	4	16	-11.3	0.9								27.0	
85	Provalata Cave	calcite coating	C2	C2X-22	-11.85	0.10	7.42	0.08																	23.9	
86	Provalata Cave	calcite coating	C2	C2N-10	-12.16	0.09	7.48	0.03	0.593	0.035	0.009	0.019	25	3	16	-11.5	0.6								23.0	
87	Provalata Cave	calcite coating	C2	C2X-23	-11.96	0.10	7.38	0.08																	19	
88	Provalata Cave	calcite coating	C2	C2X-24	-11.91	0.10	7.53	0.08																	14	
89	Provalata Cave	calcite coating	C2	C2X-25	-11.92	0.10	7.40	0.08																	8.7	
90	Provalata Cave	calcite coating	C2	C2N-3	-12.12	0.08	7.39	0.05	0.598	0.047	0.014	0.030	23	5	12	-11.8	1.0								8.5	
91	Provalata Cave	calcite coating	C2	C2X-26	-12.05	0.10	7.29	0.08																	4.5	
92	Provalata Cave	calcite marble	C3	C3X-01	-5.21	0.10	2.45	0.08																	62.3	
93	Provalata Cave	calcite marble	C3	C3X-02	-4.93	0.10	2.47	0.08																	60.4	
94	Provalata Cave	calcite marble	C3	C3-9	-4.24	0.07	2.52	0.03	0.36	0.03	0.01	0.02	164	9	12	15.500	0.900								60.3	
95	Provalata Cave	calcite marble	C3	C3X-03	-5.86	0.10	2.60	0.08																	58.5	
96	Provalata Cave	calcite marble	C3	C3-5	-3.31	0.04	2.57	0.02	0.307	0.058	0.018	0.039	230	29	11	21.7	2.1								57.4	
97	Provalata Cave	calcite marble	C3	C3X-04	-6.75	0.10	2.62	0.08																	56.5	
98	Provalata Cave	calcite marble	C3	C3-8	-3.33	0.06	2.56	0.02	0.314	0.034	0.010	0.022	219	15	12	20.900	1.200								55.4	
99	Provalata Cave	calcite marble	C3	C3X-05	-4.35	0.10	2.41	0.08																	54.5	
100	Provalata Cave	calcite marble	C3	C3X-06	-6.67	0.10	1.95	0.08																	52.1	
101	Provalata Cave	calcite marble	C3	C3X-07	-6.68	0.10	2.64	0.08																	50.2	
102	Provalata Cave	calcite marble	C3	C3X-08	-4.84	0.10	2.45	0.08																	48.5	
103	Provalata Cave	calcite marble	C3	C3-4	-8.33	0.06	3.44	0.03	0.484	0.058	0.017	0.039	70	9	11	0.2	1.4								48.4	

104	Provalata Cave	calcite marble	C3	C3X-09	-6.51	0.10	1.88	0.08																	46.6	
105	Provalata Cave	calcite marble	C3	C3X-10	-3.57	0.10	2.55	0.08																	44.7	
106	Provalata Cave	calcite marble	C3	C3X-11	-4.14	0.10	2.31	0.08																	42.9	
107	Provalata Cave	calcite marble	C3	C3-7	-5.08	0.10	2.22	0.04	0.35	0.03	0.01	0.02	171	11	12	15.20	1.10								42.2	
108	Provalata Cave	calcite marble	C3	C3X-12	-4.41	0.10	2.47	0.08																	40.6	
109	Provalata Cave	calcite marble	C3	C3X-13	-7.82	0.10	3.04	0.08																	38.7	
110	Provalata Cave	calcite marble	C3	C3-3	-8.57	0.07	4.06	0.04	0.491	0.041	0.012	0.026	67	6	12	-0.5	1.0								38.2	
111	Provalata Cave	calcite marble	C3	C3X-14	-8.51	0.10	4.66	0.08																	35.7	
112	Provalata Cave	calcite marble	C3	C3X-15	-8.63	0.10	3.04	0.08																	34.6	
113	Provalata Cave	calcite marble	C3	C3-6	-11.79	0.06	5.03	0.03	0.53	0.03	0.01	0.02	47	4	11	-7.00	0.80								34.5	
114	Provalata Cave	calcite marble	C3	C3X-16	-10.44	0.10	5.12	0.08																	32.4	
115	Provalata Cave	calcite marble	C3	C3X-17	-9.79	0.10	5.32	0.08																	30.5	
116	Provalata Cave	calcite marble	C3	C3-2	-11.71	0.02	5.74	0.02	0.565	0.033	0.010	0.021	35	4	12	-9.1	0.7								29.4	
117	Provalata Cave	calcite marble	C3	C3X-18	-10.61	0.10	5.82	0.08																	28.7	
118	Provalata Cave	calcite marble	C3	C3X-19	-10.77	0.10	5.18	0.08																	26.5	
119	Provalata Cave	calcite coating	C3	C3X-20	-11.87	0.10	7.56	0.08																	24.2	
120	Provalata Cave	calcite coating	C3	C3-1	-11.90	0.16	7.15	0.16	0.570	0.042	0.012	0.027	33	5	12	-9.7	1.0								21.7	
121	Provalata Cave	calcite coating	C3	C3-2-2	-12.15	0.06	7.30	0.04	0.622	0.042	0.011	0.024	15	3	14	-13.9	0.5	0.37	0.01	2.45	0.11	13	2.7	84	16.0	$\delta^{18}O_w$ from fluid inclusions
122	Provalata Cave	calcite coating	C3	C3-A	-11.40	0.05	6.35	0.08	0.619	0.040	0.012	0.027	16	4	11	-12.4	0.5								11.5	$\delta^{18}O_w$ from fluid inclusions
123	Provalata Cave	calcite coating	C3	C3-B	-11.01	0.10	5.68	0.07	0.614	0.047	0.014	0.032	18	5	11	-11.6	0.5								5.8	$\delta^{18}O_w$ from fluid inclusions
124	Provalata Cave	calcite coating	C3	C3-C	-11.43	0.07	6.18	0.04	0.596	0.020	0.006	0.013	24	2	12	-10.9	0.5								0.6	
125	Provalata Cave	calcite coating	PROV03	PROV03-1	-11.33	0.11	5.97	0.09	0.610	0.043	0.013	0.029	19	4	11	-11.9	0.5	0.50	0.01	1.58	0.04	20	3.5	76	92.8	$\delta^{18}O_w$ from fluid inclusions
126	Provalata Cave	calcite coating	PROV03	PROV03-2	-11.94	0.18	6.42	0.39								-13.1	0.5	0.39	0.01	1.46	0.04	22	1.9	76	65.1	$\delta^{18}O_w$ from fluid inclusions
127	Provalata Cave	calcite coating	PROV03	PROV03-3	-12.21	0.11	7.17	0.08	0.618	0.037	0.011	0.023	17	3	12	-13.2	0.8								45.9	
128	Provalata Cave	calcite coating	PROV03	PROV03-4	-12.76	0.06	7.01	0.06	0.611	0.032	0.011	0.025	19	3	9	-13.3	0.8	0.32	0.01	2.26	0.04	14	2.1	84	17.8	
129	NNW-SSE nappe	calcite marble	B05C	B05C	-3.97	0.10	2.54	0.04	0.347	0.034	0.011	0.024	175	12	10	16.7	1.2									
130	NNW-SSE nappe	calcite marble	M007C	M007C	-3.11	0.06	2.66	0.03	0.313	0.036	0.010	0.023	220	16	12	21.2	1.2									
131	NNW-SSE nappe	calcite marble	M008C	M008C	-4.01	0.12	2.60	0.04	0.394	0.035	0.009	0.018	129	7	16	12.2	0.9									
132	NNW-SSE nappe	calcite marble	M010C	M010C	-4.56	0.07	2.27	0.04	0.332	0.053	0.015	0.034	194	20	12	17.7	1.8									
133	NNW-SSE nappe	calcite marble	M006	M006	-4.01	0.10	1.85	0.08																		
134	NNW-SSE nappe	calcite marble	M007-1	M007-1	-3.39	0.10	2.42	0.08																		
135	NNW-SSE nappe	calcite marble	M007-2	M007-2	-3.63	0.10	2.49	0.08																		
136	NNW-SSE nappe	calcite marble	M008	M008	-3.54	0.10	2.37	0.08																		
137	NNW-SSE nappe	calcite marble	M009-1	M009-1	-4.69	0.10	1.64	0.08																		
138	NNW-SSE nappe	calcite marble	M010	M010	-4.58	0.10	2.14	0.08																		
139	NNW-SSE nappe	calcite marble	MEL04	MEL04	-0.04	0.10	3.73	0.08																		
140	NNW-SSE nappe	calcite marble	MEL05	MEL05	-0.76	0.10	3.62	0.08																		
141	NNW-SSE nappe	calcite marble	MEL06	MEL06	-1.12	0.10	3.89	0.08																		
142	NNW-SSE nappe	calcite marble	TR1	TR1	-1.90	0.10	2.54	0.08																		
143	NNW-SSE nappe	calcite marble	TR2	TR2	-3.70	0.10	2.75	0.08																		
144	NNW-SSE nappe	calcite marble	B03	B03	-2.27	0.10	2.61	0.08																		
145	NNW-SSE nappe	calcite marble	B05	B05	-3.56	0.10	2.53	0.08																		
146	NNW-SSE nappe	calcite marble	B08	B08	-2.33	0.10	3.13	0.08																		
147	NNW-SSE nappe	calcite marble	LP04	LP04	-1.55	0.10	3.06	0.08																		

References:

Chiodini, G., Frondini, F., Cardellini, C., Parello, F. & Peruzzi, L. Rate of diffuse carbon dioxide earth degassing estimated from carbon balance of regional aquifers: the case of central Apennine, Italy. *J. Geophys. Res.* **105**, 8423–8434 (2000)

Graham, D.W. Noble gas isotope geochemistry of mid-ocean ridge and ocean island basalts: characterization of mantle source reservoirs. *Rev. Mineral. Geochem.* **47 (1)**, 247–317 (2002)

Mook, W.G. Introduction: theory, methods, review in *Environmental isotopes in the hydrological cycle: Principles and applications* (ed. Mook, W.G.). IHP-V Technical documents in hydrology, **39 (1)**, (UNESCO, 2000)

Parkhurst, D.L. & Appelo, C.A.J. Description of input and examples for PHREEQC version 3: a computer program for speciation, batchreaction, one-dimensional transport, and inverse geochemical calculations. *US Geological Survey Techniques and Methods* **6**, 497 https://pubs.usgs.gov/tm/06/a43/ (2013)

Sano, Y. & Wakita, H. Geographical distribution of $^{3}He/^{4}He$ ratios in Japan: implications for arc tectonics and incipient magmatism. *J. Geophys. Res.* **90**, 8729–8741 (1985)

Sano, Y. & Marty, B. Origin of carbon in fumarolic gas from island arcs. *Chemical Geology*. **119 (1–4)**, 265–274 (1995)

Spötl, C. & Mattey, D. Scientific drilling of speleothems – a technical note. *International Journal of Speleology* **41**, 29-34 (2012)

Temovski, M. *et al.* Hypogenic origin of Provalata Cave, Republic of Macedonia: a distinct case of successive thermal carbonic and sulfuric acid speleogenesis, *International Journal of Speleology* **42(3)**, 235–264 (2013)

Zheng, Y-F. Carbon–oxygen isotopic covariation in hydrothermal calcite during degassing of CO_2. *Mineralium Deposita* **25**, 246–50 (1990)
